# Network analysis of 18 attention-deficit/hyperactivity disorder symptoms suggests the importance of “*Distracted*” and “*Fidget*” as central symptoms: Invariance across age, gender, and subtype presentations

**DOI:** 10.3389/fpsyt.2022.974283

**Published:** 2022-10-21

**Authors:** Lu Liu, Yi Wang, Wai Chen, Yuan Gao, Haimei Li, Yufeng Wang, Raymond C. K. Chan, Qiujin Qian

**Affiliations:** ^1^Peking University Sixth Hospital/Institute of Mental Health, Beijing, China; ^2^National Clinical Research Center for Mental Disorders, Key Laboratory of Mental Health, Ministry of Health (Peking University), Beijing, China; ^3^Neuropsychology and Applied Cognitive Neuroscience Laboratory, CAS Key Laboratory of Mental Health, Institute of Psychology, Beijing, China; ^4^Department of Psychology, University of Chinese Academy of Sciences, Beijing, China; ^5^Mental Health Service, Fiona Stanley Hospital, Perth, WA, Australia; ^6^Curtin Medical School, Curtin University, Perth, WA, Australia; ^7^Curtin enAble Institute, Curtin University, Bentley, WA, Australia; ^8^Graduate School of Education, University of Western Australia, Perth, WA, Australia; ^9^School of Medicine, University of Notre Dame Australia, Fremantle, WA, Australia; ^10^School of Psychology, Murdoch University, Perth, WA, Australia

**Keywords:** ADHD, symptom structure, network analysis, community, subgroups

## Abstract

The *network theory of mental disorders* conceptualizes psychiatric symptoms as networks of symptoms that causally interact with each other. Our present study aimed to explore the symptomatic structure in children with attention-deficit/hyperactivity disorder (ADHD) using network analyses. Symptom network based on 18 items of ADHD Rating Scale-IV was evaluated in 4,033 children and adolescents with ADHD. The importance of nodes was evaluated quantitatively by examining centrality indices, including Strength, Betweenness and Closeness, as well as Predictability and Expected Influence (EI). In addition, we compared the network structure across different subgroups, as characterized by ADHD subtypes, gender and age groups to evaluate its invariance. A three-factor-community structure was identified including inattentive, hyperactive and impulsive clusters. For the centrality indices, the nodes of “*Distracted*” and “*Fidget*” showed high closeness and betweenness, and represented a bridge linking the inattentive and hyperactive/impulsive domains. “*Details*” and “*Fidget*” were the most common endorsed symptoms in inattentive and hyperactive/impulsive domains respectively. On the contrary, the “*Listen*” item formed a peripheral node showing weak links with all other items within the inattentive cluster, and the “*Loss*” item as the least central node by all measures of centrality and with low predictability value. The network structure was relatively invariant across gender, age and ADHD subtypes/presentations. The 18 items of ADHD core symptoms appear not equivalent and interchangeable. “*Distracted*” and “*Fidget*” should be considered as central, or core, symptoms for further evaluation and intervention. The network-informed differentiation of these symptoms has the potentials to refine the phenotype and reduce heterogeneity.

## Introduction

Attention-deficit/hyperactivity disorder (ADHD) is conceptualized as a neurodevelopment disorder, characterized by age-inappropriate levels of inattention, hyperactivity and impulsivity associated with impairments across settings. According to the DSM-5 (1) and ICD-11 taxonomic systems (2), ADHD is a polythetic condition of which all affected individuals share similar characteristics but are non-identical in specific patterns of symptom expression (3). In DSM-5, diagnostic threshold for children is set at 6 or more inattentive symptoms, and/or, 6 or more hyperactive/impulsive symptoms; whereas in adults, the threshold is at 5. None of the 18 diagnostic criteria is designated as “essential” or ranked as “more essential” for diagnostic classification. Two children may therefore meet the diagnostic threshold for ADHD with two substantially different sets of symptoms, for instance with overlap of only three symptoms in each domain, equating to 50% non-overlap. In adults, the overlap can be further reduced to one symptom in each domain, equating to about 89% non-overlap. Therefore phenotypic heterogeneity can be substantial across cases.

To address phenotypic heterogeneity, one current available method is to subclassify caseness based on one of the three “presentations” defined by DSM-5, such as “combined,” “predominantly inattentive,” or “predominantly hyperactive/impulsive” presentations. Within these subcategories, all symptoms are still weighted equally without hierarchical ranking, while the patterns of interaction and co-occurrence amongst symptoms are considered as irrelevant—aside the threshold of symptom count. Contrasting with the assumptions made by DSM-5 and ICD-11, empirical findings indicate the contrary to be true. A recent study reported that 18 items contribute independently to the clinical diagnosis of ADHD, with some of them carrying more weight (4). Moreover, these 18 core symptoms may not occur in the same frequency in one particular ADHD sample, and especially across samples (5). The simple sum of the symptoms (assumed to be equally-weighted)—as advocated by DSM-5 and ICD-11—will lose important clinical information and introduce greater clinical and etiological heterogeneity. In doing so, such a diagnostic approach may reduce treatment effectiveness as well as encumbering research in unraveling the pathogenesis and etiology of ADHD.

Several efforts have been conducted to overcome these limitations and provide more informative approaches to identify the latent architecture of ADHD symptomology. These include factor analyses (6, 7) and item response theory (IRT) (3, 8, 9). However, both factor analytical and IRT approaches cannot evaluate how items connect and trigger one another, especially, how they influence and perpetuate a disorder through their interactions with other symptoms within the symptom network. Such shortcomings can be addressed by a different approach that is based on the *network theory of mental disorders*, which conceptualizes psychiatric symptoms as networks of symptoms that causally interact (10). The traditional “latent variable approach” views the manifestations of observable symptoms (i.e., 18 ADHD diagnostic symptoms) arising from the latent construct of ADHD that cannot be measured directly (i.e., the underlying common cause of a clinical disorder). In contrast, network models of psychopathology view a set of symptoms as a causal system, initiating, perpetuating and maintaining a disorder through mutual influences without a latent causal driver (11). The findings from a network analysis can be therefore interpreted as revealing a causal system (11), within which some symptoms connect and interact with some more than others (12), exerting differential influences within the network.

Several recent studies have attempted to establish the cross-sectional network models of ADHD symptoms. Martel et al. (13) performed the first network analyses to describe interactions of ADHD symptoms across age windows. Their results indicated age-related differentiation of ADHD symptoms over the development. As a child matures, most symptoms change their expression, except “*easily distracted*,” which remains unchanged while serving as an important bridge symptom between inattentive and hyperactive/impulsive domains. More specifically, “e*asily distracted*” and “*difficulty sustaining attention*,” unlike others, remain central symptoms across developmental periods. In a cross-sectional data, Silk et al. (5) found that “*easily distracted*” and “*does not listen*” were the most frequently endorsed inattentive symptoms, and “*interrupts*” was the most frequently hyperactive/impulsive symptom in the ADHD group. Within their network of ADHD symptoms, the hyperactive/impulsive domain was organized into two smaller subclusters with “*awaiting turn*” as a bridge”—indexing “betweenness.” However, inattentive symptoms conformed to two general clusters, around (i) planning/organization (*disorganized*, *loses things*, *forgetful*) and (ii) sustaining attention (*distracted*, *difficulty sustain attention*, *fails close attention*, *avoids mental effort* and *not follow instructions*). Of these, “*lose things*” was the node with the highest “betweenness centrality.” In addition, “*does not listen*” was found to be mostly isolated (5). Preszler and Burns (14) found that “*concentration difficulties*” showed the highest “expected influence.” Goh et al. (15) examined the network of ADHD and sluggish cognitive tempo symptoms, and identified eight bridge items between ADHD and sluggish cognitive tempo networks; and these items were more efficient indicators of impairment than the simple pool of all items. Taken together, these studies have identified unique characteristics of different items as described by network analysis parameters, despite inconsistencies across studies.

Several factors which may account for inconsistencies in the aforementioned findings, such as sample heterogeneity introduced by ADHD subtypes (termed “presentations” in DSM-5), gender and age distribution (16, 17). Would sample characteristics, and variable symptom distributions influence the network structure of ADHD across studies? While Martel et al. (13) assessed ADHD symptoms across age range, their findings were based on visual assessment and not statistical evaluation, given visual inspection based on subjective judgment may reduce reproducibility of their findings (12). In another study, no significant differences in network structure and bridge impairment items were found across samples of children and adolescents (13), but subthreshold cases and non-ADHD comparisons were analyzed together as a single group in this study. There are significant methodological limitations in network analysis studies of ADHD. Furthermore, to our knowledge, no study in the extant literature has examined the potential influence of subtype and gender.

The present study aimed to evaluate the network structure of ADHD symptoms in a large clinical sample of Chinese Han participants with ADHD (age 6-16), in order to address the aforementioned gaps in the literature. In particular, it aimed to probe for evidence against the equal-weight and interchangeability assumptions of ADHD symptoms. We hypothesized that (1) some specific central symptoms should be identified, that is DSM-5 symptoms are not equivalent in terms of centrality as parameterized by “strength,” “closeness,” “betweenness,” and “expected influence” and (2) the network structure across different subgroups, as characterized by ADHD subtypes (i.e. presentations), gender and age-groups, would be invariant.

## Materials and methods

### Participants

Children and adolescents with ADHD were recruited from Peking University Sixth Hospital/Institute of Mental Health. ADHD, subtypes and comorbidity were classified based on a semi-structured diagnosed interview conducted by a child psychiatrist was conducted using the Clinical Diagnostic Interview Scale (CDIS); (18) according to the DSM-IV (19) criteria. Moreover, all participants met the following inclusion criteria: (1) age between 6 and 16 years; (2) drug-naïve; and (3) with estimated full-scale IQ above 70. Exclusion criteria included a history of neurological disorders and other psychiatric disorders. The present study was approved by the Ethics Committee of Peking University Institute of Mental Health. Written consent was obtained from the parents and guardians of the participants.

### Measures

ADHD Rating Scale-IV (ADHD RS-IV) was used to assess the psychopathology of ADHD. It was rated by parent or primary carer with best knowledge of the child on a 4-point Likert-type scale (1 = not at all, 2 = sometimes, 3 = often, and 4 = always). It consists of 18 symptom items according to the DSM-IV criteria for ADHD.

Nine inattentive symptoms were: (1) *Fails to give close attention to details or makes careless mistakes in schoolwork*, coded as “***Details***”; (2) *Has difficulty sustaining attention in tasks or play activities*, coded as “***Span***”; (3) *Does not seem to listen when spoken to directly*, coded as “***Listen***”; (4) *Does not follow through on instructions and fails to finish work*, coded as “***Follow***”; (5) *Does not follow through on instructions and fails to finish work*, coded as “***Finish***”; (6) *Avoid tasks (e.g., schoolwork, homework) that require sustained mental effort*, coded as “***Avoid***”; (7) *Loses things necessary for tasks or activities*, coded as “***Loss***”; (8) *Is easily distracte*d, coded as “***Distracted***”; (9) *Is forgetful in daily activities*, coded as “***Forget***”.

Nine hyperactive/impulsive symptoms were coded as: (1) *Fidgets with hands or feet or squirms in seat*, coded as “***Fidget***”; (2) *Leaves seat in classroom or in other situations in which remaining seated is expected*, coded as “***Seat***”; (3) *Runs about or climbs excessively in situation in which it is inappropriate*, coded as “***Run***”; (4) *Has difficulty playing or engaging in leisure activities quietly*, coded as “***Noisy***”; (5) *Is “OnTheGo” or acts as if “driven by a motor”*, coded as “***OnTheGo***”; (6) *Talks excessively*, coded as “***Talk***”; (7) *Blurts out answers before questions have been completed*, codes as “***Blurt***”; (8) *Has difficulty waiting turn*, coded as “***Wait***”; (9) *Interrupts or intrudes on others*, coded as “***Interrupt***.”

#### Network estimation and network inference

Symptom network based on 18 items of ADHD RS-IV was evaluated in participants with ADHD. The nodes were core symptoms of ADHD. The edges of the network were partial correlations between each pair of nodes after controlling for all the other nodes in the network. As our data were ordinal in nature, Gaussian Graphical Model was estimated using graphical Least Absolute Shrinkage and Selection Operator (gLASSO) (20) in combination with Extended Bayesian Information Criteria (EBIC) model selection (21). The tuning parameter, set as 0.5 (21, 22), was applied to shrink the partial correlation coefficients to obtain a stable network. The placement of the nodes in the network was determined by the force-directed Fruchterman–Reingold algorithm (23). The R software (version 4.0.0, available at https://cran.r-project.org/) was used for network analysis in this study, R-package “qgraph” (24) was used for network estimation.

The importance of nodes was further evaluated quantitatively by examining centrality indices, including *strength*, *betweenness*, and c*loseness* (11), as well as *predictability* and *expected influence* (EI). A node with high betweenness means it occupies frequently in the shortest paths between pairs of the other nodes. High closeness indicates close connections with all the other nodes in the network. Strength is calculated by summing the absolute weights of all the edges connected to the node. To account for negative edges that suppress other nodes contrasting with positive edges that activate other nodes, EI assesses the strength of a node accounting for the negative edges (25). In addition, the Predictability of a node measures the extent to which a node can be predicted by all the other nodes in the network (26). The higher predictability indicates the high controllability of the node, that it could be controlled *via* its neighboring symptoms in the network. The standardized z scores of centrality indices were calculated, higher z score indicates greater relative importance of a given node in the network. The ‘‘qgraph’’ and ‘‘mgm’’ packages^1^ implemented in R statistical software were used for centrality calculation and visualization.

#### Community detection

Two different methods were adopted to detect the community of the ADHD symptom network in the current study. Firstly, the more conventional walktrap algorithm was used to detect community using Exploratory Graph Analysis *via* the R-package ‘‘EGAnet’’.^2^ This method does not allow cross-loading, which means one item can only belong to one community. However, we considered the possibility that a few ADHD symptoms may have relatively high loading on more than one community. Thus, Clique Percolation algorithm was also used to detect communities allowing items assigned to more than one community in the network, *via* the R-package ‘‘Clique Percolation’’.^3^

#### Network comparisons

In order to examine the variance or invariance of ADHD symptom networks across ADHD subtypes (ADHD-I vs. ADHD-C; ADHD-HI was not included for network comparisons due to its limited sample size), genders (males vs. females) and among four age groups (6–7 years, 8–9 years, 10–11 years, and 12 years old or over; the subjects with 12 years old or over were not further devided at 2-year interval due to the limited sample size in the older age groups), we estimated these networks in samples stratified by these parameters respectively; and examined the invariance of network structure, global strength, and edge-weights using two-tailed permutation tests (10,000 times) (27). The test for invariance of network structure depends on the maximum difference between matrices consisting of all connections. The global strength of a network, defined as the sum of the absolute edge-weights of all pairs of nodes, was calculated and compared. False discovery rate (FDR) correction was adopted to address multiple comparisons of edge-weights, and adjusted *p* values were calculated using the Benjamini and Hochberg (28) method implemented in R. The ‘‘Network Comparison Test’’ package^4^ implemented in R statistical software was used for the network comparisons. The significance threshold was set at *p* < 0.05 for the invariance test of network structure and global strength, and adjusted *p* < 0.05 for differences on edge-weights.

#### Network stability and accuracy

The stability of each estimated network, including the accuracy of edge-weights and centrality were evaluated according to the method published by Epskamp, Borsboom and Fried (29). Firstly, the accuracy of edge-weights was estimated by drawing non-parametric bootstrapped Confidence Intervals (CIs) with 2,500 permutations. Narrow bootstrapped CIs denote low sampling variability in edge-weights, indicating an accurate network. Secondly, we investigated the stability of strength using case-dropping subset bootstrap to assess how well the order of centralities was retained in only portions of data. The CS-coefficient was used to measure the maximum drop in proportions to retain a correlation of 0.7 in at least 95% of the sample. It is suggested that the CS-coefficient should preferably above 0.5 in order to render the network stable. Thirdly, we conducted bootstrapped difference tests between edge-weights that were non-zero and node strength of the ADHD symptoms. The ‘‘bootnet’’ package^5^ implemented in R statistical software was used to estimate the network stability.

## Results

A total of 4,033 children and adolescents with ADHD (3,355 boys and 678 girls) were recruited for analyses; and their demographic and clinical characteristics are summarized in Table 1.

**TABLE 1:** The demographic and clinical characteristics of children with ADHD.

	ADHD (*N* = 4,033)
**Gender (*n*,%)**	
Males	3,355 (83.2)
Females	678 (16.8)
Age in Month (Mean ± SD)	118.4 ± 31.2
FSIQ (Mean ± SD)	103 ± 15
**DSM-IV subtypes (n,%)**	
ADHD-I	1,876 (46.5)
ADHD-HI	167 (4.1)
ADHD-C	1,627 (40.3)
**ADHD symptoms (Mean ± SD)**	
Inattentive	18.1 ± 4.1
Hyperactive/impulsive	13.5 ± 6.0
Total	31.5 ± 8.2
**Comorbidities (*n*,%)**	
ODD	1,047 (25.9)
CD	140 (3.5)
Mood Disorders	168 (4.2)
Anxiety Disorders	412 (10.2)
Tic Disorders	594 (14.7)
Learning Disorders	1,374 (34.1)

ADHD, attention-deficit/hyperactivity disorder; FSIQ, full-scaled intelligence quotient; SD, standard deviation; DSM-IV, Diagnostic and Statistical Manual of Mental Disorders 4th edition; ADHD-I, ADHD inattentive type; ADHD-HI, ADHD hyperactive-impulsive type; ADHD-C, ADHD combined type; ODD, oppositional defiant disorder; CD, conduct disorder; Mood disorder includes major depression disorder, dysthymic disorder and bipolar disorder; Anxiety disorder includes specific phobia, social phobia, obsessive-compulsive disorder, generalized anxiety disorder and separation anxiety disorder.

### Description of 18 attention-deficit/hyperactivity disorder symptom items

Table 2 summarizes the mean rating scores and the frequencies of endorsement for each symptom in ADHD participants. For the frequency distribution of each item, we found “*Details*” as the most frequently endorsed symptom followed by “*Distracted*” in the attentive domain, while “*Fidget*” as the most frequently endorsed symptom followed by *“OnTheGo*” in the hyperactive/impulsive domain.

**TABLE 2:** The mean rating scores and the frequencies of endorsement for each symptom in the entire sample and subgroups of ADHD.

Items	Rating score (Mean ± SD)	Total (%) (*n* = 4,033)	Rank	Ranks in Silk et al. (5)	Subtypes (%)			Gender (%)		Age-groups (%)			
							
					ADHD-I (n = 1,876)	ADHD-HI (n = 167)	ADHD-C (n = 1,627)	Males (*n* = 3,355)	Females (*n* = 678)	6–7 years (*n* = 1,136)	8–9 year (*n* = 1,264)	10–11 year (n = 642)	≥12 year (*n* = 907)
**Inattentive**													
**Details**	2.29 ± 0.60	92.7*	**1**	9 (closeatt)	94.3*	**74.3**	**93.4**	92.6*	93.5*	**87.9**	93.8*	95.0*	96.3*
Span	2.15 ± 0.67	86.6	3	4 (susatt)	87.0	67.1	88.8	85.8	90.1	86.2	86.5	87.5	86.2
Listen	1.86 ± 0.73	71.5	6	2 (listen)	69.2	58.7	76.7	71.1	73.0	75.8	72.9	69.3	66.8
Follow	2.10 ± 0.72	83.2	5	3 (instruct)	84.9	55.1	85.2	83.8	80.7	81.1	84.1	84.3	84.2
Finish	1.67 ± 0.82	61.8	8	6 (org)	60.9	35.9	66.9	62.0	60.5	65.6	61.3	62.5	57.6
Avoid	2.21 ± 0.76	85.3	4	5 (avoid)	87.5	50.3	87.3	85.4	84.8	81.1	86.1	86.4	88.8
Loss	1.79 ± 0.79	64.5	7	7 (loses)	65.1	29.3	68.0	64.3	65.3	64.1	65.0	64.3	64.8
**Distracted**	2.30 ± 0.64	**92.3**	**2**	1 (distract)	**91.5**	86.8*	94.6*	**92.2**	**93.2**	92.8*	**93.4**	**92.1**	**91.2**
Forget	1.69 ± 0.77	60.3	9	8 (forget)	61.1	24.6	64.2	60.6	58.8	58.3	58.6	63.1	63.8
**Hyperactive**													
**Fidget**	2.04 ± 0.87	77.2*	**1**	2 (fidget)	65.2*	91.0*	90.2*	78.8*	69.6*	86.3*	80.3*	74.1*	65.5*
Seat	1.12 ± 0.93	33.6	9	3 (seat)	17.9	49.1	50.8	35.2	25.5	44.8	33.5	25.9	25.4
Run	1.24 ± 0.95	40.8	7	5 (runs)	21.1	62.3	61.2	43.1	29.6	53.7	41.8	29.1	32.6
Noisy	1.54 ± 0.90	56.2	3	9 (quiet)	35.6	79.6	77.4	57.8	48.2	68.1	59.3	49.4	43.2
**OnTheGo**	1.75 ± 1.00	**65.4**	**2**	7 (motor)	**47.8**	**89.2**	**82.9**	**66.5**	**60.2**	**77.8**	**69.9**	**61.1**	48.7
**Impulsive**													
Talk	1.60 ± 0.97	56.1	4	6 (talks)	38.3	75.4	73.6	56.7	53.2	61.7	55.3	56.1	**50.9**
Blurt	1.53 ± 0.93	53.0	5	8 (blurts)	35.4	68.9	71.5	54.2	47.1	59.4	53.3	52.5	45.8
Wait	1.19 ± 0.94	37.3	8	4 (turn)	19.5	53.9	55.9	39.1	28.3	43.5	36.1	36.3	31.8
Interrupt	1.46 ± 0.90	50.4	6	1 (interrupt)	30.2	76.6	71.0	52.0	42.0	60.0	50.4	46.1	42.9

The items in bold indicated the top two frequent symptoms in inattentive and hyperactive/impulsive domains, while the * indicated the highest item. The underlined items indicated the symptoms with obvious different frequency between our study and that in Silk et al. (5).

This pattern across respective subgroups, based on subtypes, gender or age groups, was similar generally. However, some minor anomalies deviated from the overall pattern were noted. For the hyperactive/impulsive domain, “*Fidget*” and “*OnTheGo*” were the most frequently endorsed items, except for the 12-years group in which “*Talk*” was the second most endorsed symptom. As predicted by developmental maturity, the endorsement of hyperactive symptoms declined with age.

### Network structure of attention-deficit/hyperactivity disordersymptom network

**FIGURE 1:**
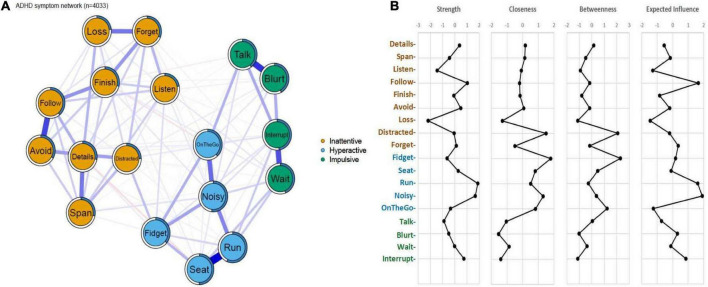
**(A)** Symptom network of ADHD core symptoms. The blue edges indicate positive partial correlations and edges in red indicates negative partial correlations. Thicker lines represent stronger connections. The blue ring around each node represents its predictability values. **(B)** Centrality indices and Expected Influence of the symptom network in ADHD. Standardized z-scores of strengths, closeness, betweenness and expected influence for each node in the network were plotted.

By visual inspection, an overall three-factor-community structure was identified including inattentive, hyperactive and impulsive clusters. Yet, it was possible to differentiate the hyperactive and impulsive items to form two subgroups within one hyperactive/impulsive cluster. The symptom “*Talk*,” which bridged hyperactive and impulsive clusters, was more closely linked with impulsive symptoms, rather than hyperactive symptom – a pattern more aligned with ICD-10 conceptualization (which classifies “*Talk*” as an impulsive item) than DSM classification. Notably, the edge-connection between “*Distracted*” and “*Fidget*” represented a bridge linking inattentive and hyperactive/impulsive domains (Figure 1A).

For the centrality indices (Figure 1B), the nodes of “*Distracted*” in inattentive domain and “*Fidget*” in the hyperactive/impulsive domain showed the highest closeness and betweenness, suggesting that they are closely connected to the other nodes in the network, and also act as “bridges” between clusters. The “*Run*” and “*Noisy*” nodes in the hyperactive/impulsive domain showed relatively high strength in the network, and after taking in account the negative values of partial correlation coefficients, the nodes of “*Run*,” “*Noisy*,” and “*Follow*” showed high degrees of expected influence, indicating that these nodes exert strong influences on the other nodes in the network. The “*Loss*” symptom from the inattentive cluster was the least central node by all measures of centrality. The mean predictability value of all modes was 37.0%. Among them, the predictability value of “*Listen*” in the inattention cluster was the lowest (22.2%), which was consistent with its diffuse but weak links with other items as shown in Figure 1A. The highest predictability value was indicated for the “*Run*” item in the hyperactive/impulsive domain (56.6%), which might be mainly due to its strong connection with “*Seat*” and “*Noisy*” as shown in Figure 1A. No significant correlation was found between the mean rating scores and the network properties (*r* = 0.316, *p* = 0.201 for Betweenness; *r* = 0.282, *p* = 0.257 for Closeness; *r* = −0.204, *p* = 0.417 for Strength; *r* = −0.218, *p* = 0.386 for Expected Influence), indicating that the network parameters were more influenced by the rating level.

### Communities of attention-deficit/hyperactivity disordersymptom network

**FIGURE 2:**
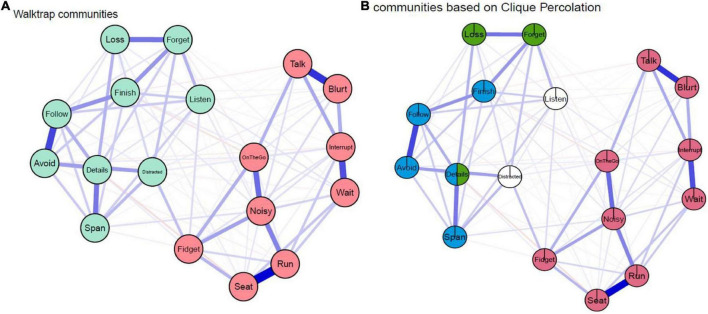
Communities detected by **(A)** Walktrap algorithm: The R packages “EGAnet” was used to detect communities in the network and two communities were identified. **(B)** Clique Percolation algorithm: K was set as three as the algorithm requires a minimum k of 3 and I was set as 0.15 to obtain the highest ratio threshold and a large χ threshold. The R package “Clique Percolation” was used.

By applying the walktrap algorithm, we quantitatively identified two communities, which was partly consistent with the theoretical factor models of 18 ADHD symptoms, where the 18 items were parcellated into (i) the “*Inattentive*” factor as one community and (ii) the merged “*Hyperactive*/*Impulsive*” factor as the second community (Figure 2A). However, this community detention method did not allow cross-loading, which meant that one item could only belong to one community in the network. Thus, we applied the Clique Percolation method to detect communities and the results showed three communities in the network, but the nodes in the inattentive domain separated and cohered in an unconventional manner. Notably, all nine “*Hyperactive*/*Impulsive*” items form its respective factor; but *“Finish,” “Follow,” “Span,”* and *“Details”* formed the second community, whilst *“Loss,” “Forget,”* and *“Details”* formed the third community. “*Listen*” and “*Distracted*” were not assigned to any community (Figure 2B). In other words, the inattentive nodes separated into two inattentive clusters, while “*Listen*” and “*Distracted*” were unassigned and “*Details*” was assigned to two communities.

### Network structural invariance across “age,” “gender,” and “ADHD subtypes” groups

**FIGURE 3:**
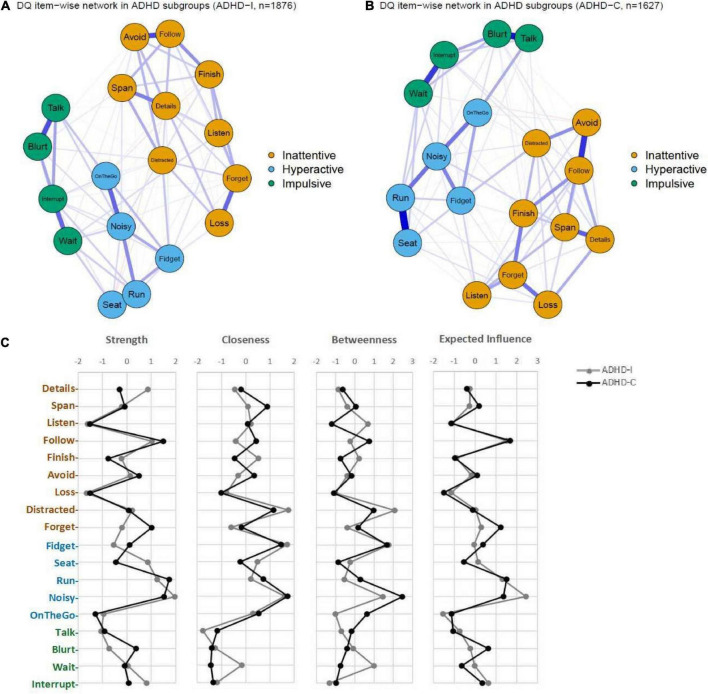
The network structures in different subtypes. **(A)** network in ADHD-inattentive subtype (ADHD-I); **(B)** network in ADHD-combined subtype (ADHD-C); **(C)** centrality indices and Expected Influence of the symptom networks in ADHD-I and ADHD-C.

We compared the ADHD symptom networks between ADHD-I and ADHD-C subtypes to test the invariance of network structures, global strength and edge-weights. The results showed that ADHD-I and ADHD-C subtypes had similar symptom network structures (*M* = 0.09; *p* = 0.482). A nominally stronger global strength was found in ADHD-C than ADHD-I (7.63 vs. 7.22; *S* = 0.41, *p* = 0.011) (Figure 3). However, no significant difference in edge-weights was found after FDR correction. In other words, the networks were at large invariant across ADHD subtypes.

**FIGURE 4:**
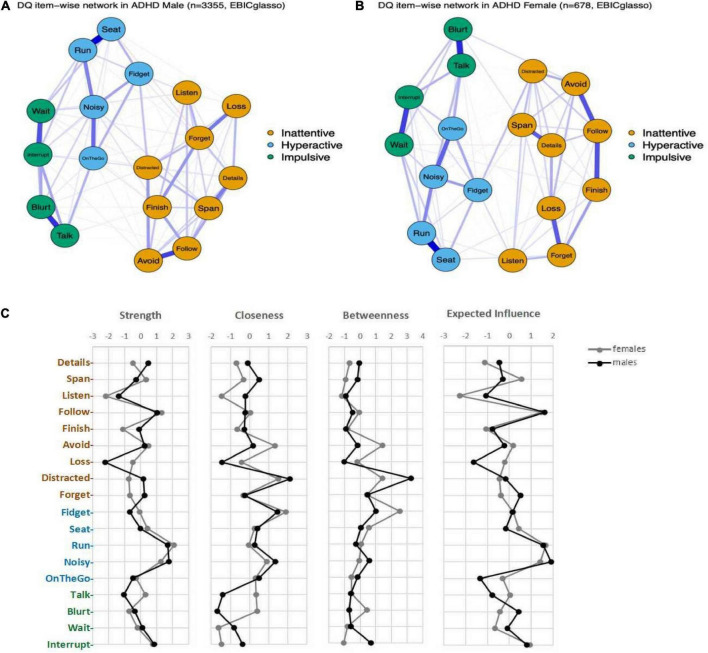
The network structures in males and females. **(A)** network in males with ADHD; **(B)** network in females with ADHD; **(C)** Centrality indices and Expected Influence of the symptom networks in males and females.

No significant differences were found across male and female groups, indicative of the invariance of network structure (*M* = 0.12, *P* = 0.137) or global strength (*S* = 0.31, *p* = 0.339) (Figure 4). To minimize the potential bias caused by gender imbalance in sample size, we further conducted network comparisons between females and subsamples of male participants (100 times resampling with 1,000 iterations). The results indicated that only 14 and 4 out of 100 times subsampling showed significant difference between males and females for the invariance of network structure and global strength, respectively. In other words, no significant difference was found between symptom networks of males and females.

**FIGURE 5:**
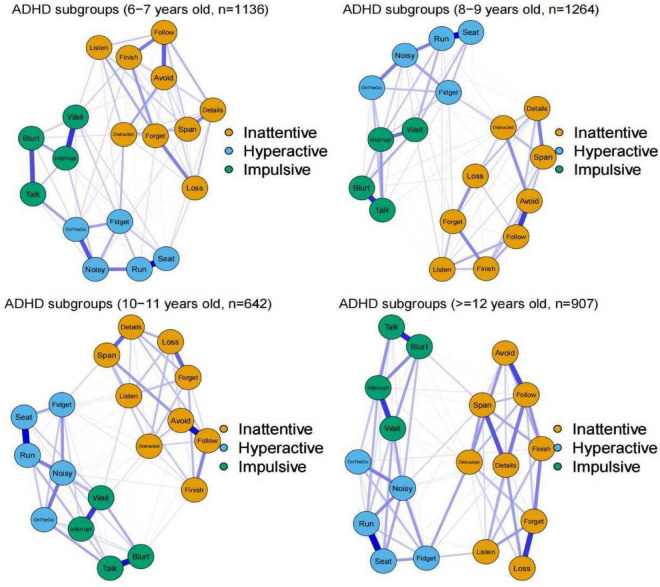
The network structures in different age groups. The blue edges indicate positive partial correlations and edges in red indicates negative partial correlations. Thicker lines represent stronger connections.

Network structure invariance across age groups was estimated for four age strata (age 6–7 stratum; 8–9 stratum; 10–11 stratum; and ≥12 stratum) within the whole sample (Figure 5). The results indicated comparable network structure across these four strata, and no significant differences were found in terms of the network structure invariance (*p*-values > 0.1) and global strength invariance (*p*-values > 0.5) when all permutations of pairing among four strata were compared. Our detailed analyses suggested that the ADHD symptom network structures are consistent across age spanning childhood to adolescent (6–16 years old in this sample).

### Network stability

Stability analysis for each estimated network (whole sample, gender groups, ADHD subtypes, age groups) showed relatively narrow bootstrapped CIs, suggesting reliable edge-weights. All the CS-coefficients for strength [CS (cor = 0.7)] were larger than 0.5, which suggests that the centrality indices were quite stable. More details of the results on network stability can be found in the Supplementary material.

## Discussion

There were three key findings in our study. Firstly, a three-factor-community structure was identified for 18-item ADHD core symptoms, including inattentive, hyperactive and impulsive domains. Interestingly, “*Talk*” showed strong links with impulsive symptoms, which is more in accordance with the designation in ICD-10. However, the community detected using the Clique Percolation method suggested two sub-clusters in inattentive domain, implicating potential novel substructure or a chance finding or an artifact arising from the Clique Percolation method. In our findings, *“Finish,” “Follow,” “Span,”* and *“Details”* formed the one *inattentive* community, whilst *“Loss,” “Forget,”* and *“Details”* formed a different *inattentive* community. “*Listen*” and “*Distracted*” were not assigned to any community. To our knowledge, no such inattentive substructure has been reported before. Secondly, the two most commonly endorsed symptoms, “*Distracted*” and “*Fidget*” (for inattentive and hyperactive/impulsive domains respectively), showed important bridge effects, which represented critical nodes for further evaluation. Thirdly, as hypothesized, the network structure was relatively invariant across ADHD subtypes, gender and age, indicating its robustness for generalization.

In this study, a three-factor-community structure was identified; however, the hyperactive/impulsive cluster could be also further parcellated in two respective subgroups. This is consistent with the two- or three- factor structure of ADHD yielded by exploratory and confirmatory factor analyses (30). Notably, within the hyperactive/impulsive cluster, “*Talk*” (classified as a hyperactive symptom by DSM systems) showed stronger links with impulsive symptoms, and not with hyperactive symptoms, which is more in accordance with the designation in ICD-10 (3, 31). Interestingly, when items were freed permitting to belong to more than one community, a totally novel structure of three communities were identified, with the conventional inattentive symptoms divided into three groups: *“Finish,” “Follow,” “Span,” “Avoid,”* and “*Details*” forming the first inattentive community; *“Loss,” “Forget,”* and *“Details”* forming the second; and “*Listen*” and “*Distracted*” not assigned to any community. The node “*Details*” was assigned to two communities. These are empirically derived findings, which are in line with previous findings yielded by different analytical approaches. As reported in previous factor analyses, both the “*Distracted*” and “*Listen*” were found to be high cross-loading items (6, 7). Our results are therefore in line with previous findings in the literature, especially the “*Distracted*” item playing the “bridge” role which links the inattention and hyperactivity/impulsivity domains as shown in this study.

The two clusters identified in inattentive domain with “*Details*” as overlap node, suggesting the potential heterogeneity in inattention dimension. For the sub-cluster containing “*Loss*,” “*Forget*,” and *“Details*” items, the “*Loss*” item showed the lowest centrality for strength, closeness, betweenness and EI, and the centrality indices of “*Forget*” were also not strong. We speculate that the nodes in this sub-cluster may occur in participants with higher levels of inattention.

For the frequency distribution of each item, we found “*Details*” as the most frequently endorsed symptom followed by “*Distracted*” in the attentive domain, while “*Fidget*” as the most frequently endorsed symptom followed by *“OnTheGo*” in the hyperactive/impulsive domain. The symptoms frequencies in our study indicated some differences from that reported by Silk et al. (5) (shown in Table 2). In the inattentive domain, they reported “*Distracted*” as the most frequently endorsed symptom followed by “*Listen*.” Strikingly, the highest ranked inattentive symptom in our sample was “*Details*” (92.7%, ranked 1st); in contrast, it was the lowest (61.6%, ranked 9th) in the study reported by Silk et al. (5). The “*Listen*” symptom (71.5%) was ranked 6th in our sample, which was much lower than that in Silk et al. (91.1%, ranked 2nd). For the hyperactive/impulsive domain, the difference seems were more extensive. The most frequently endorsed symptom reported by Silk et al. (5) was “*Interrupt*” (84.2%, ranked 1st) followed by “*Fidget*” (76%, ranked 2^nd^). In our findings, “*Fidget*” was also found to be common (77.2%, ranked 2nd); however, the “*Interrupt*” was with lower frequency (50.4%, ranked 6th). Other differences were noted for “*Seat*” (Ranked 9th in our study vs. Ranked 3rd in Silk et al.), “*Noisy*” (Ranked 3rd vs. Ranked 9th), “*OnTheGo*” (Ranked 2nd vs. Ranked 7th) and “*Wait*” (Ranked 8th vs. Ranked 4th) between these two studies.

These aforementioned differences of symptom endorsement might help to explain the discrepancy of network properties in some extent. For instance, “*Distracted*” showed strong correlation with “*Fidget*” representing the only one bridge between inattentive and hyperactive/impulsive domains, implicating potential critical roles of this symptom in spreading and maintaining its influences between the two domains. This finding supported for the previous finding that “*Distracted*” cross-loads to the hyperactive/impulsive factor (6). Moreover, the results indicated “*Distracted*” as the highest endorsed inattentive symptom in ADHD-HI subtype and “*Fidget*” as the highest endorsed hyperactive/impulsive symptom in ADHD-I subtype, which also support the bridge effects of these two items in some extent. “*Distracted*” and “*Fidget*” were both most frequent symptoms in inattentive and hyperactive/impulsive domains respectively in both our study (irrespective of ADHD of subtypes) and also that of Silk et al. (5). Strikingly, they were the least severe symptoms, suggesting that they were endorsed even at low inattentive or hyperactive/impulsive levels) (32). Evidence from analysis based on IRT also indicated that “*Distracted*” was the most informative parent- and teacher-rated ADHD symptom with relatively lower severity threshold (3, 8, 32). In addition, “*Distracted*” is one of the three DSM-5 items used for screening in the research gold-standard diagnostic instrument—“kiddie-schedule for affective disorders and schizophrenia-present and lifetime version” (K-SADS-PL) (33). Taken together, “*Distracted*” and “*Fidget*” symptoms appear to be the most central symptoms in ADHD psychopathology. These two symptoms may represent critical therapeutic targets - as an intervention, which target on any one symptom or their connection can effectively reduce the symptoms severity and/or their influences within the network, should augment treatment effect and promote functional recovery. We should note that, the item “*Details*,” with the highest frequency of endorsement in our sample, was not with high network properties, except for the relatively high Strength. In addition, it was also the item showing the most marked discrepancy in endorsement rate between our study (92.7%, ranked 1st) and that in Silk et al. (61.6%, ranked 9th). It would be very interesting to investigate such difference, which might arise from cross-cultural difference (34).

Our findings also identified some items with low centrality indices and/or expected influence. For inattentive symptoms, “*Listen*” showed diffuse but weak links with other symptoms in both inattentive and hyperactive/impulsive domains, and the lowest predictability value. In the community detected using the Clique Percolation method, the node “*Listen*” was not assigned to any community. In the existing literature, evidence from factor analyses has indicated a weaker mean factor loading of “*Listen*,” weaker than that of any other inattentive symptoms (6, 7). “*Listen*,” also showed high cross-loading with the hyperactive/impulsive factor (7). Silk et al. (5) found that “*Listen”* occupied a peripheral and isolated position in the identified network, a finding similar to ours—despite “*Listen*” being the most frequently endorsed inattentive symptoms. If such findings were further replicated, future revision of diagnostic criteria may consider replacing “*Listen*” with another more informative item. In addition, “*Loss*” from the inattentive cluster was the least central node by all measures of centrality in our study and with low predictability value. This finding markedly differs from those reported previously that “*Loss*” was the node with the highest betweenness centrality although this symptom appears to be in the periphery of the network as represented in network (5). However, a recent IRT analysis reported “*Loss”* as a “difficult item” (i.e., endorsed only in severe ADHD cases), which is therefore present only in those individual embodying higher level of ADHD latent traits (32). In other words, a higher level of inattention is required for “*Loss*” to manifest clinically, which corroborates our findings.

Our identified network was at large invariant across the subgroups of age, gender and ADHD subtypes. In addition, the endorsement of each item was also largely consistent across these groups. Martel et al. (13) reported that the network became more differentiated from preschool childhood to adulthood (age spanning from 3 to 36) in a population sample. In contrast, our present study found that the networks were comparable across different age groups in the clinical diagnosed ADHD sample of a narrower age range (6–16 years old). This indicated that the interconnectivities and interactions of symptom items in the ADHD symptom networks of our clinical sample were consistent over different developmental windows from childhood to adolescence (6–16 years old). However, we should note that Martel et al. did not conduct formal community analyses and did not compare the different communities in age groups. The age effects reported by them were based on visual inspection. Consistent with the evidence from the existing literature (17), the frequencies of symptom endorsement in our present study indicated some difference among age groups, which suggested that the clinical expression of each specific symptom in different developmental periods should be different. However, we have reflected further over these findings, and note that the symptom expression difference among age groups may not be equivalent to the variance of symptom network. For example, as indicated in Table 2, while all hyperactive/impulsive symptoms declined with age, “*Fidget*” remains the most frequently endorsed item. This just reflected the invariance and stability of symptom network, rather than invariance in symptom expression. Similar invariance of network structure has also been reported in previous studies (15). In fact, a recent IRT study indicated that the hierarchical structure of ADHD symptoms was invariant across age and gender (9), which also supported our present findings. It should be noted that we have combined the subjects with 12 years old and over for group comparisons due to the limited sample size from the statistical perspective. Puberty, as an important developmental period, may be with strong change in behaviors. However, based on the identified invariance across age, gender and ADHD subtype, we propose that even with more refined sub-grouping, the network structure should not change. As for adulthood, it should be explained with caution, since there are some controversies on the definition of adults with ADHD as persistent ADHD, adult-onset ADHD or late-diagnosed ADHD (35). Nevertheless an alternative explanation should be also mentioned that our present study consisted of a cross-sectional sample of different age cohorts; and it is not a longitudinal follow-up study in which the developmental trajectories of symptoms over age can be tracked. The age effect is therefore confounded with the cohort effect. Moreover, all the children with ADHD came to the clinics for intervention because of functional impairments. This means that they were roughly in the same disease stage or severity – due to referral bias (case ascertainment bias). Therefore, such bias may contribute toward network invariance. So referral bias and cohort effect may lead to such artifacts, which can only be overcome by studies of longitudinal design. For the subtype, we did not find significant difference in network structure between ADHD-I and ADHD-C. Although the ADHD-HI subtype was not included for analyses and group comparisons, we anticipated that its network structure should be also invariant. By the age increasing, the prevalence of ADHD-HI would significantly decline. The invariance across age groups found in our analyses may support our assumption of the invariant network in ADHD-HI. Definitely, further collection of cases with the diagnosis of ADHD-HI subtype/presentation would help us to elucidate this more precisely. Subtypes, gender and age groups have been suggested to be potential confounding factors in ADHD studies, which would increase the sample heterogeneity (36). The invariance of networks structure across subtypes, gender and age groups give us some hints that future investigation of the underlying pathophysiology of certain symptoms, such as the widespread symptoms and/or some important “bridge” symptoms, might be expected to reduce the above-mentioned heterogeneity.

Our present findings illustrated the potential network structure of ADHD symptoms based on the network theory of mental health – that challenges the current assumptions. The current conceptualizations of ADHD within DSM-5 and ICD-11 systems are bound by three assumptions: the first is *equal-weight assumption*, the second is *latent common cause assumption* and the third is *interchangeability assumption*. Emerging evidence suggests that all three assumptions may not hold. In addition to the previous results from both factor and IRT analyses, the network analyses also provide evidence against the equal-weight assumption (5). Importantly, the findings from network analysis including our present study also provided preliminary evidence against both the common cause and interchangeable assumptions. More specifically, network theory provides an alternative model to *latent common cause* model in conceptualizing psychopathogenesis; and postulates specific phases in the pathogenic process. In the *asymptomatic phase*, the network is dormant. In the *activation phase*, an external event activates the initial cluster of symptoms, which are then spread to activate other connected (but hitherto dormant) symptoms within the network. In the *maintenance phase*, the network becomes self-sustaining and self-perpetuating once fully activated; and the psychopathology become stuck in an active state in the network, despite the removal of the initial triggering external event; and characteristically, the removal of the initial trigger does not lead to recovery as the pathological network has now become autonomous (i.e., independent of the initial trigger) (10). Overall, the incremental spread across nodes within a network should be directional and causal. Although our present study of cross-sectional data could not address this dynamic process, future studies using longitudinal data from normal subjects, sub-threshold cases shading with ADHD cases may compute and map the temporal progression of networks over developmental windows, which could help to understand the dynamic changes and psychopathogenic process of ADHD.

Our present findings have some clinical implications. First, both DSM-5-TR and ICD-11 utilize the polythetic system for disease classification. In the current diagnostic criteria of ADHD, none of the 18 diagnostic criteria is designated as “essential” or ranked as “more essential” for diagnostic classification. Our findings suggest that not all the ADHD 18 items should be evaluated in equal weight. For example, “*Distracted*” and “*Fidget*” might need to be given higher weight than others, while inversely for “*Listen*” and/or “*Loss*.” Instead of summing all items together equally, exploration on whether some key symptoms or their connections might refine the phenotype to decrease the heterogeneity, such as the use of weighted symptoms counts as more informative phenotypes for the investigation of the neurobiological underpinnings of ADHD (5). A previous study using IRT analysis reported “*Loss*” as a “difficult item,” suggesting this item embodying greater “weight” in ADHD latent traits (32). IRT analyses, however, are predicated upon the latent trait assumption. In contrast, network theory of psychopathology posits that symptoms propagate within a network and activate other nodes; and the theoretical emphasis is therefore different. In the network analysis, the nodes of early activation and acting as bridges between clusters are potential key targets for early intervention, as deactivation of these nodes can prevent further propagation and downstream activation of disease network; and therefore they can be weighted as being critical in prevention. The notion of “weight” is therefore used differently to denote different kinds of significance between IRT (i.e., severe latent traits) and network analysis (i.e., critical early activation nodes). These weighted symptoms would be the important target for precise intervention (37), albeit “weight” can be used in different sense between these two different paradigms. Second, if replicated, our preliminary findings could also become relevant to intervention strategies targeting critical nodes within the networks. Target intervention of the central, or core, symptoms or their connections may promote greater efficiency of ADHD treatments, promoting symptomatic (and by inference functional) improvements. To our knowledge, applying network analysis to elucidate treatment effects has not been explored in the literature of ADHD, whereas some promoting evidence has been yielded in the study of other psychiatric disorders (38). Future studies can either re-analyze existing data of treatment studies or target new designs of treatment studies evaluating changing configurations of symptom networks during pre and post intervention windows. Third, the identification of these key symptoms in ADHD network might be also valuable for the etiological study, as the potential target variables for the exploration of the more precise neurobiological mechanisms. For example, we can just focus on the “*Distracted*” symptom and investigate its related brain alteration, cognitive impairment and the genetic background. Recently, some researchers have attempted to combine the symptomatic data with the neuropsychological (39) or genetic data (40) to construct the network. The brain imaging data could also be used for similar analyses. Another possible way is to explore the relationship between the identified core symptom severity and the structural and/or functional features of the hub brain regions. Finally, more work is needed to elucidate the network structures of ADHD symptoms, including introducing other features such as emotional domains and common comorbidities, as empirical elucidation and refinement of symptom structures may also advance the progress of ADHD nosology (41).

This study has several limitations. First, as mentioned above, our analyses were based on cross-sectional data. Therefore, we could not examine the dynamic changes of the network structure, and therefore could not explore the dynamic relationship between symptoms in the network overtime and development. Longitudinal data could help to illustrate the dynamic change of ADHD symptom network over developmental windows to avoid age cohort effects by using cross-sectional data of participants from different age groups, as in our study and other published studies. Second, we only included 18 items of ADHD symptoms in our analyses based on the current DSM-5 criteria. In addition to the inattentive, hyperactive and impulsive domains, other important features such as emotion dysregulation, internalizing and externalizing symptoms are potential informative candidates for inclusion in the future analyses, given emotion dysregulation symptoms have been proposed to be a core component of ADHD symptoms (42), or sentinel features demarcating ADHD “complex” and “simplex” subtypes (43). Third, certain comorbidities were not excluded in our present study, and the extents to which existing common comorbidities may influence the network patterns or vice versus were adjusted as covariates in our analyses. Future network analyses, however, may probe the direct causal structure within network of comorbidity patterns (12). Fourth, we did not include normal controls in our present study. According the dynamic model proposed by Borsboom (10), the network structure and/or properties are likely to be different between the neurotypical subjects and the clinical cases, which was verified in a recent study of social anxiety disorder (44). Indeed, the recruitment of normally developed controls in the future could help to explore whether the network structures detected in the clinical samples can be replicated in the general population, or the network properties are different and distinct in neurotypical subjects. Moreover, a large longitudinal follow-up of a general population would help to identify the naturalistic dynamic development of ADHD symptoms, ranging from neurotypical to sub-threshold cases, shading into those with a clinical diagnosis of ADHD; yet such a study would be very large and ambitious in both scale and cost, beyond those available in our current study. The observed dynamic features would promote the identification of the key symptoms for preventive and early intervention. Finally, we have used a large sample to construct the network, which could guarantee the stability and the accuracy. Further replication in an independent sample would help to validate our findings and promote the understanding of the network structure of ADHD symptoms.

## Conclusion

Our present study utilized network analysis to identify novel symptom structures in children with ADHD. Our findings confirm the invariance of symptom networks across age, gender and ADHD subtypes, and suggest “*Distracted*” and “*Fidget*” to be the core symptoms for ADHD. The network-informed differentiation of ADHD symptoms highlights the potential to refine the phenotype and reduce heterogeneity in this clinical group.

## Data availability statement

The raw data supporting the conclusions of this article will be made available by the authors, without undue reservation.

## Ethics statement

The studies involving human participants were reviewed and approved by Peking University Sixth Hospital/Institute of Mental Health. Written informed consent to participate in this study was provided by the participants’ legal guardian/next of kin.

## Author contributions

LL, YiW, QQ, and RC conceptualized the idea and designed the study. LL and HL organized the database. LL, YiW, WC, YG, and YuW analyzed and interpreted the findings and wrote up the drafts of the manuscript. RC and QQ interpreted the findings and commented the drafts critically. All authors contributed to manuscript revision, read and approved the submitted version.

## Footnotes

1
https://CRAN.R-project.org/package=qgraph

2
https://CRAN.R-project.org/package=EGAnet

3
https://CRAN.R-project.org/package=CliquePercolation

4
https://CRAN.R-project.org/package=NetworkComparisonTest

5
https://CRAN.R-project.org/package=bootnet
