# Supplementary material for: Network analysis of 18 attention-deficit/hyperactivity disorder symptoms suggests the importance of “Distracted” and “Fidget” as central symptoms: Invariance across age, gender, and subtype presentations
Source: Front Psychiatry. 2022 Oct 21;13:974283. doi: 10.3389/fpsyt.2022.974283 (PMC9633674; doi:10.3389/fpsyt.2022.974283)
Supplement: Supplementary file 1 [file Data_Sheet_1.docx]

Supplementary Material

## The results for network stability and accuracy in the whole sample


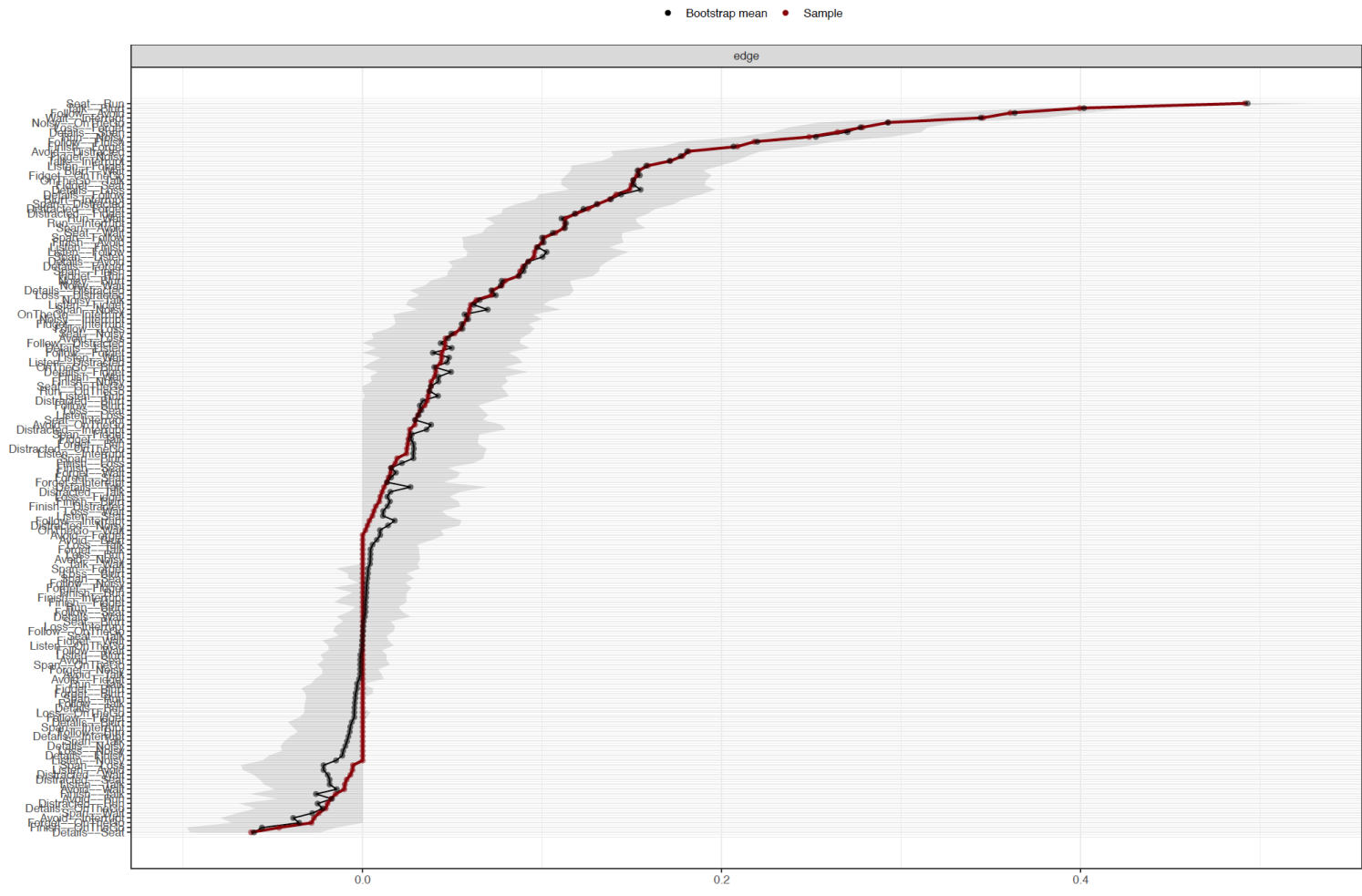


**Figure S1**. Bootstrapped CIs of estimated edge-weights for the network in whole sample. The red line indicates the sample values and the grey area indicates the bootstrapped CIs. Each horizontal line represents one edge of the network ordered by edge-weights.


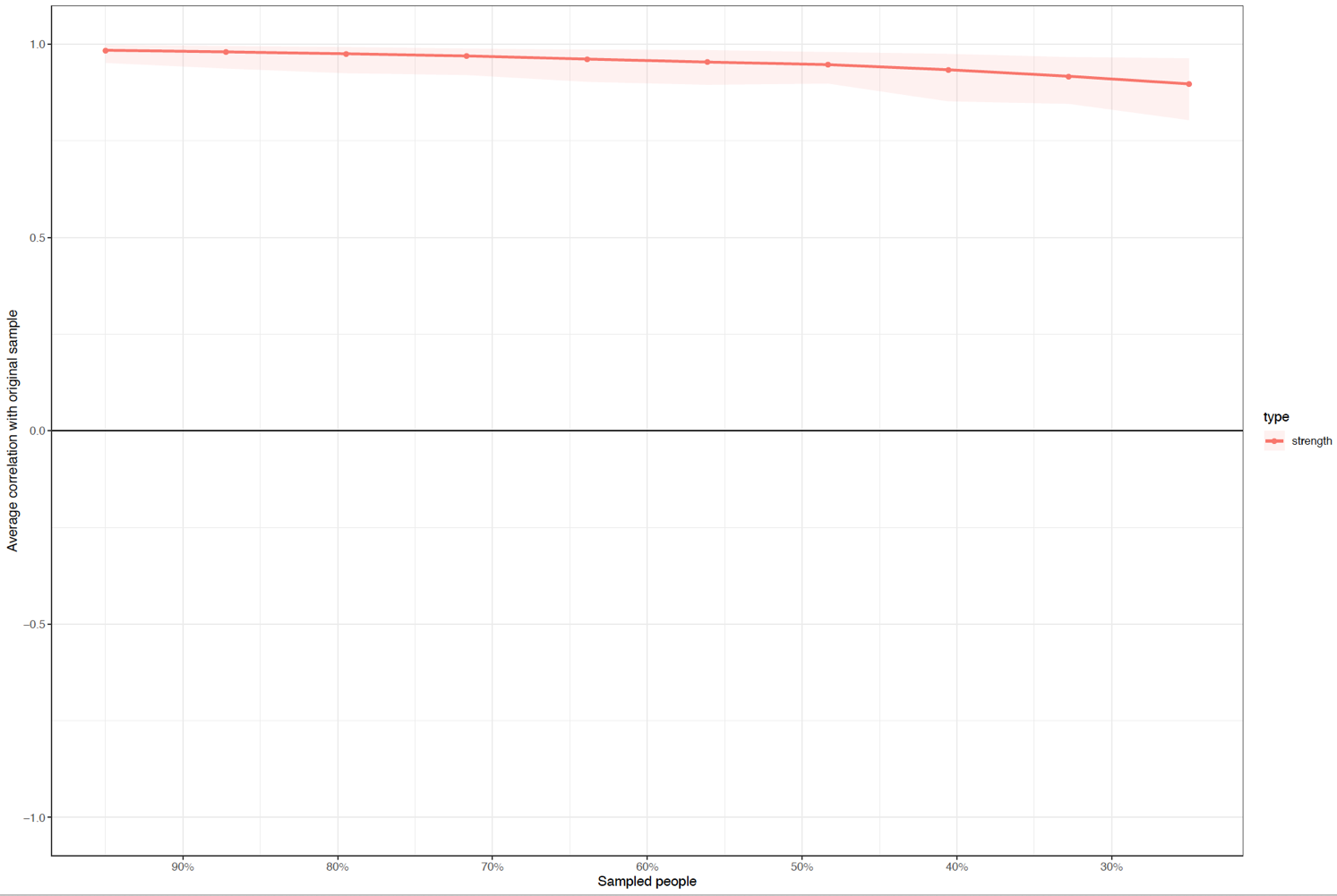


**Figure S2. Average correlations between strengths of networks estimated with sampled participants and original sample.** Lines indicate the means and areas indicate the range from the 2.5^th^ to the 97.5^th^ percentile.


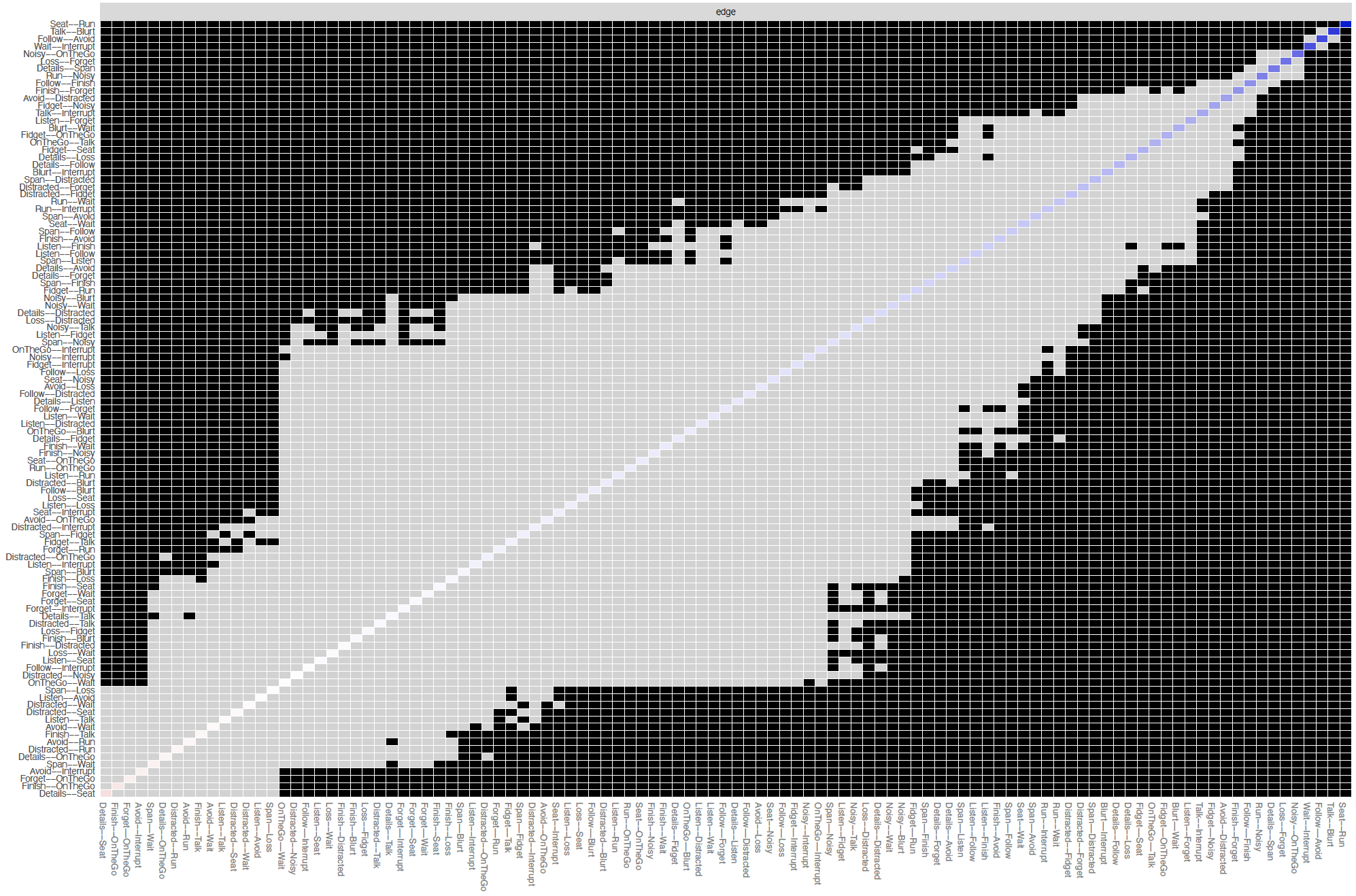


**Figure S3. Bootstrapped difference tests on the non-zero edge-weights of the estimated network.** Black boxes indicate edges that differed significantly from another corresponding edges in the matrix. Coloured boxes in the edge-weight plot correspond to the colour of edges in the estimated network.


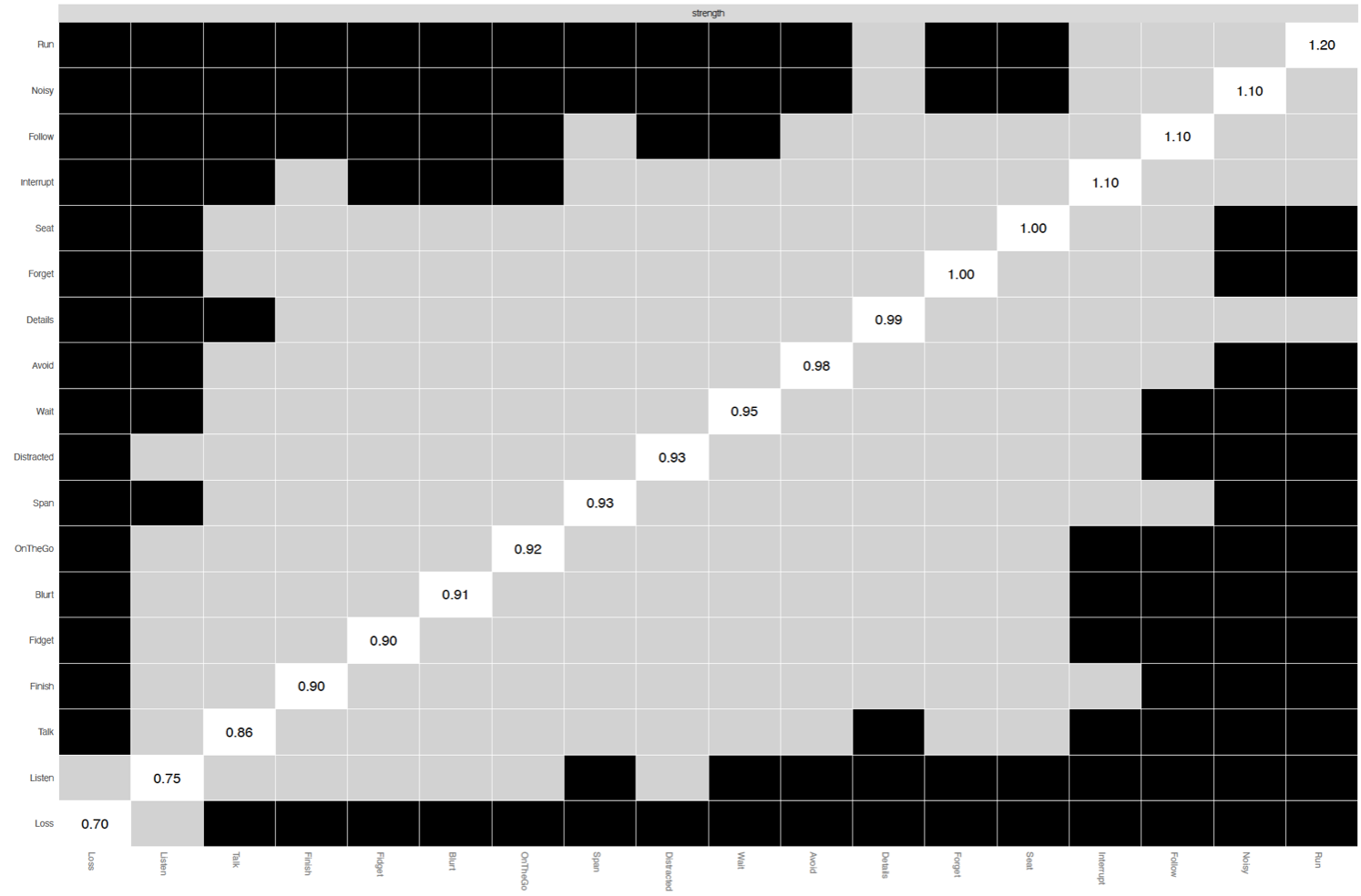


**Figure S4. Bootstrapped difference tests on the nodal strength of all the variables in the network.** Black boxes indicate nodes that differed significantly from another corresponding node in the matrix. Numbers in white boxes in the centrality plot show the strength of the corresponding node.

## The results for network stability and accuracy for male participants


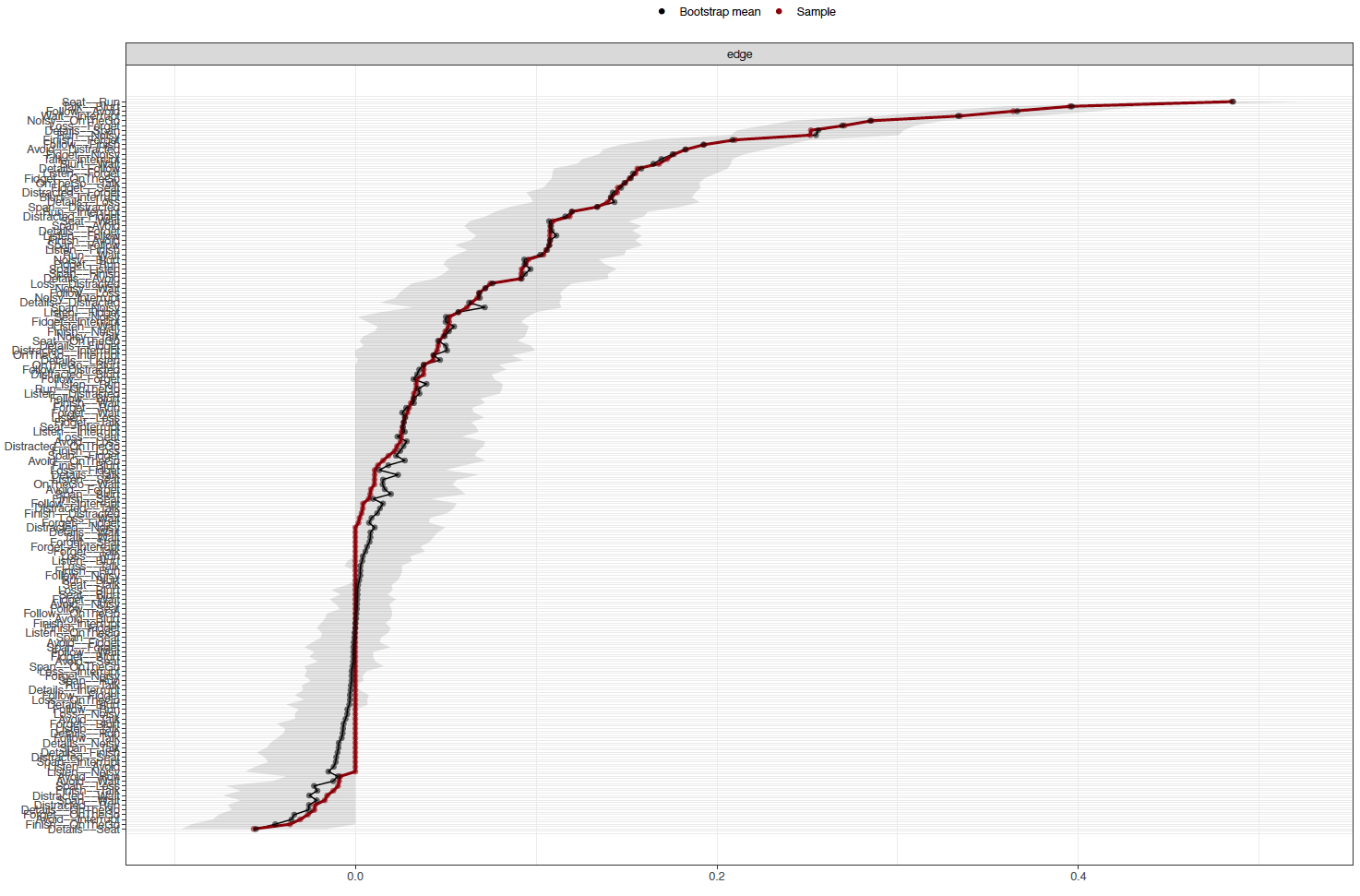


**Figure S5**. **Bootstrapped CIs of estimated edge-weights for the network.**


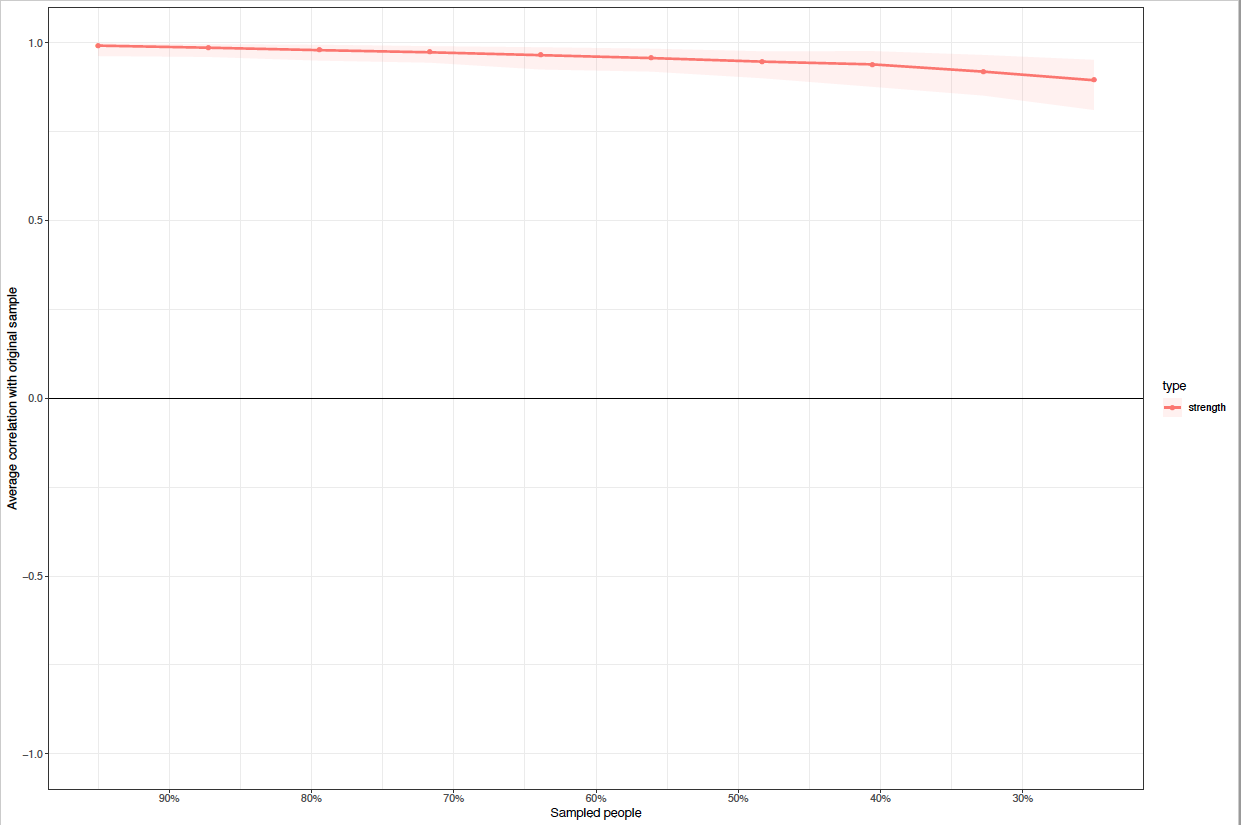


**Figure S6. Average correlations between strengths of networks estimated with sampled participants and original sample.**


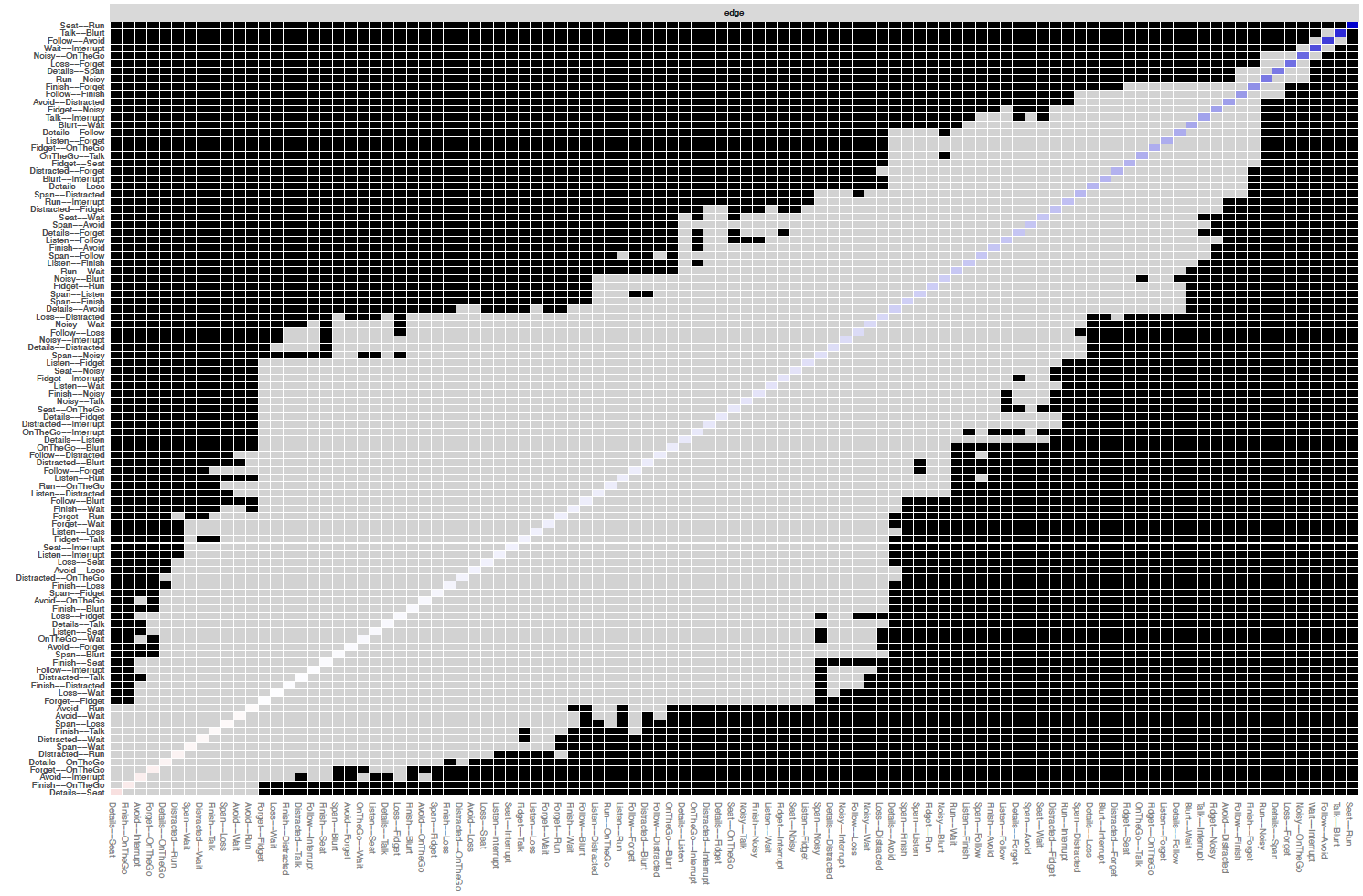


**Figure S7. Bootstrapped difference tests on the non-zero edge-weights of the estimated network.**


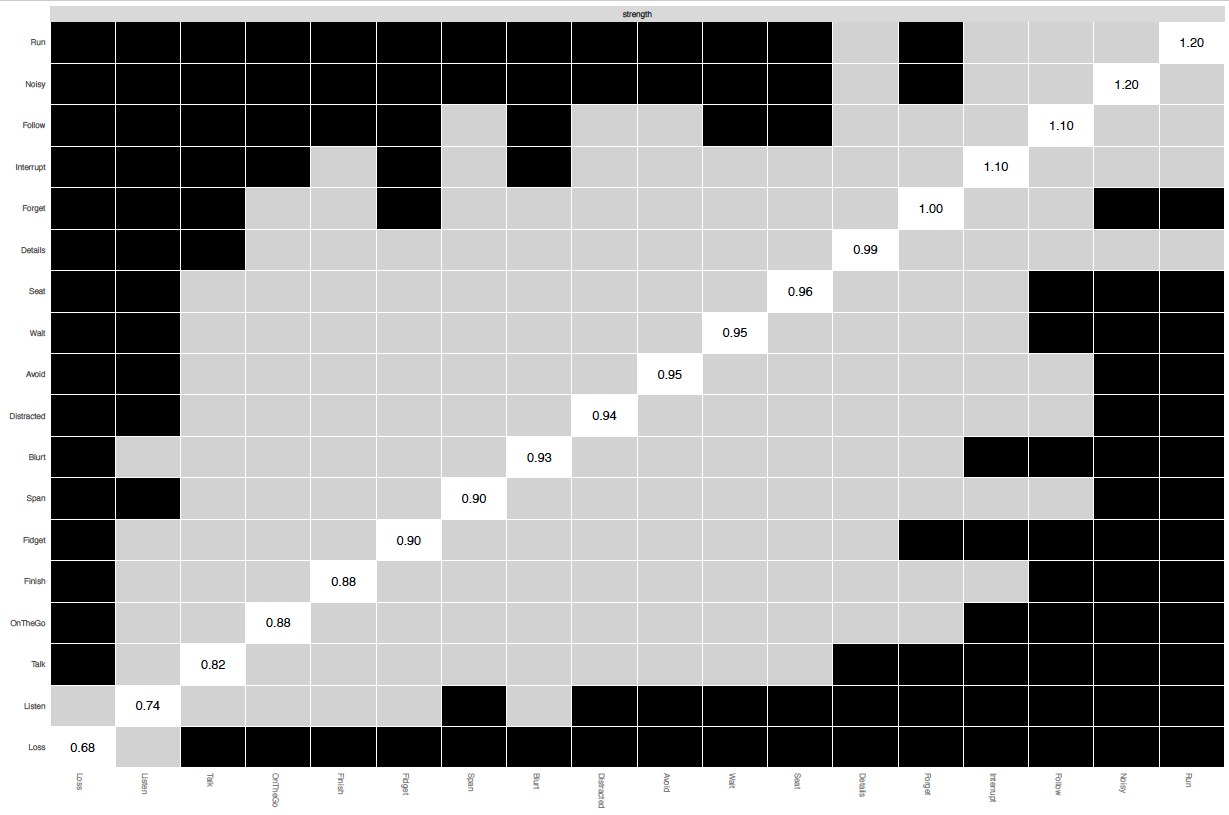


**Figure S8. Bootstrapped difference tests on the nodal strength of all the variables in the network.**

## The results for network stability and accuracy for females


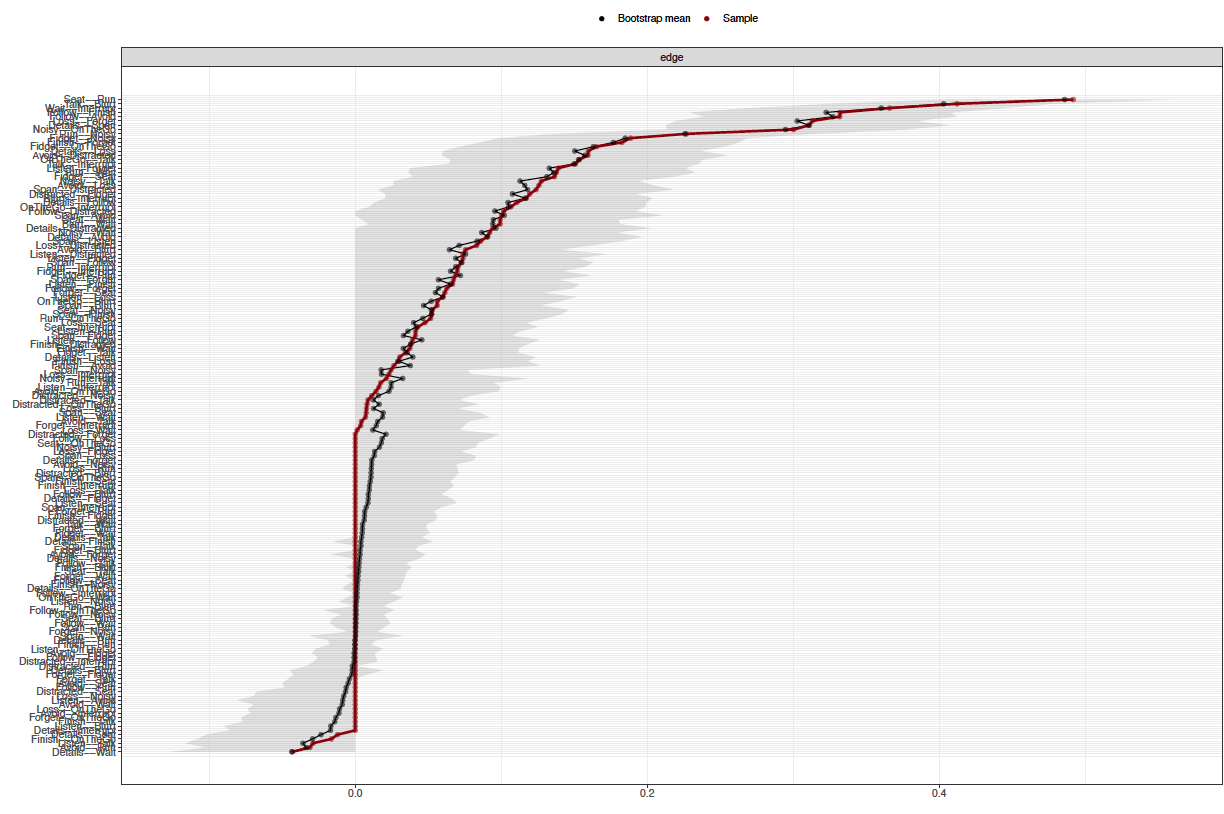


**Figure S9. Bootstrapped CIs of estimated edge-weights for the network.**


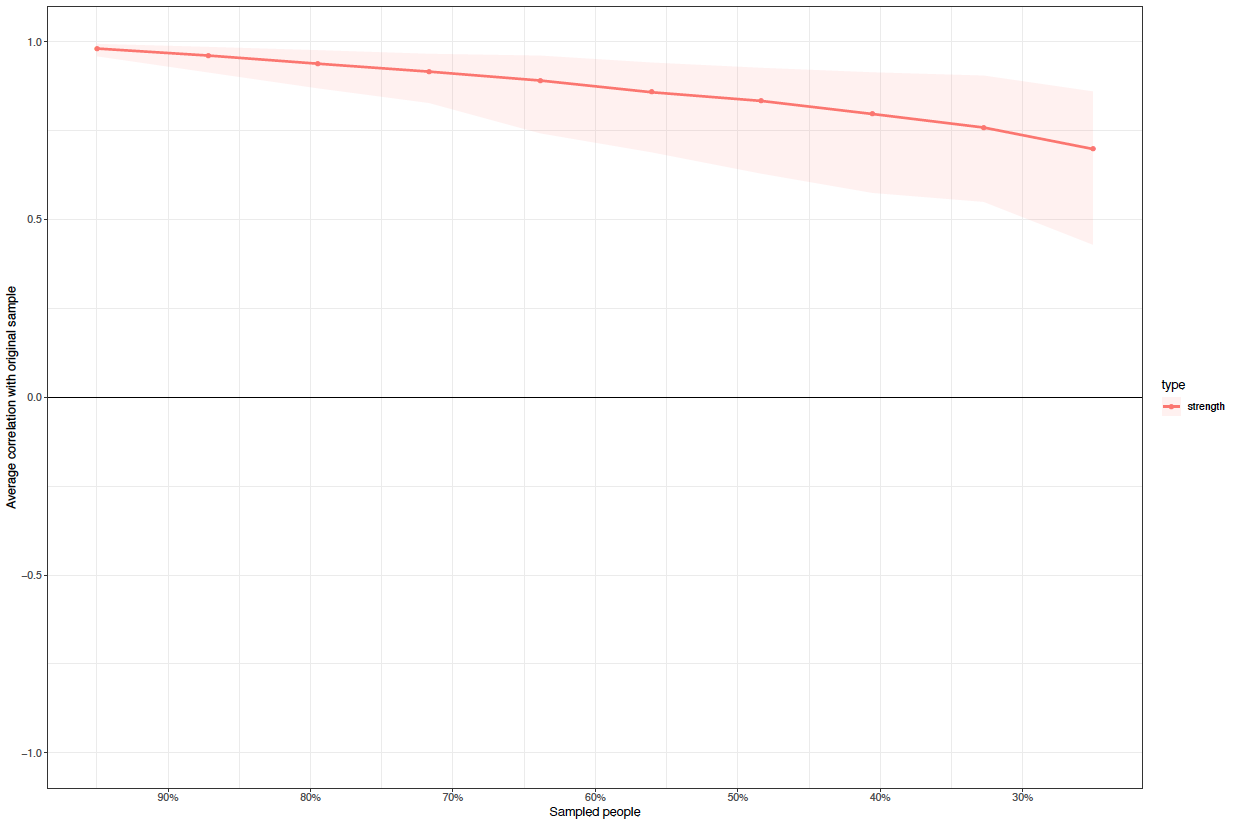


**Figure S10. Average correlations between strengths of networks estimated with sampled participants and original sample.**


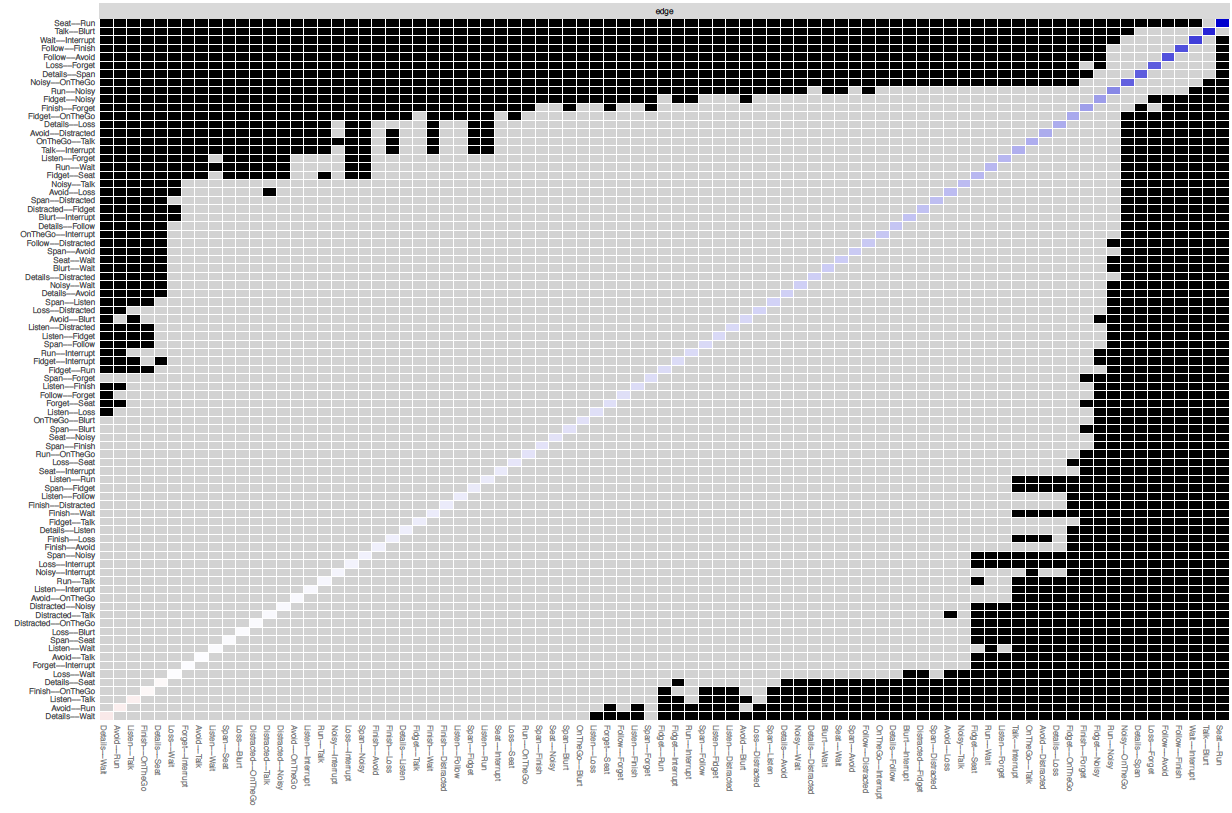


**Figure S11. Bootstrapped difference tests on the non-zero edge-weights of the estimated network.**


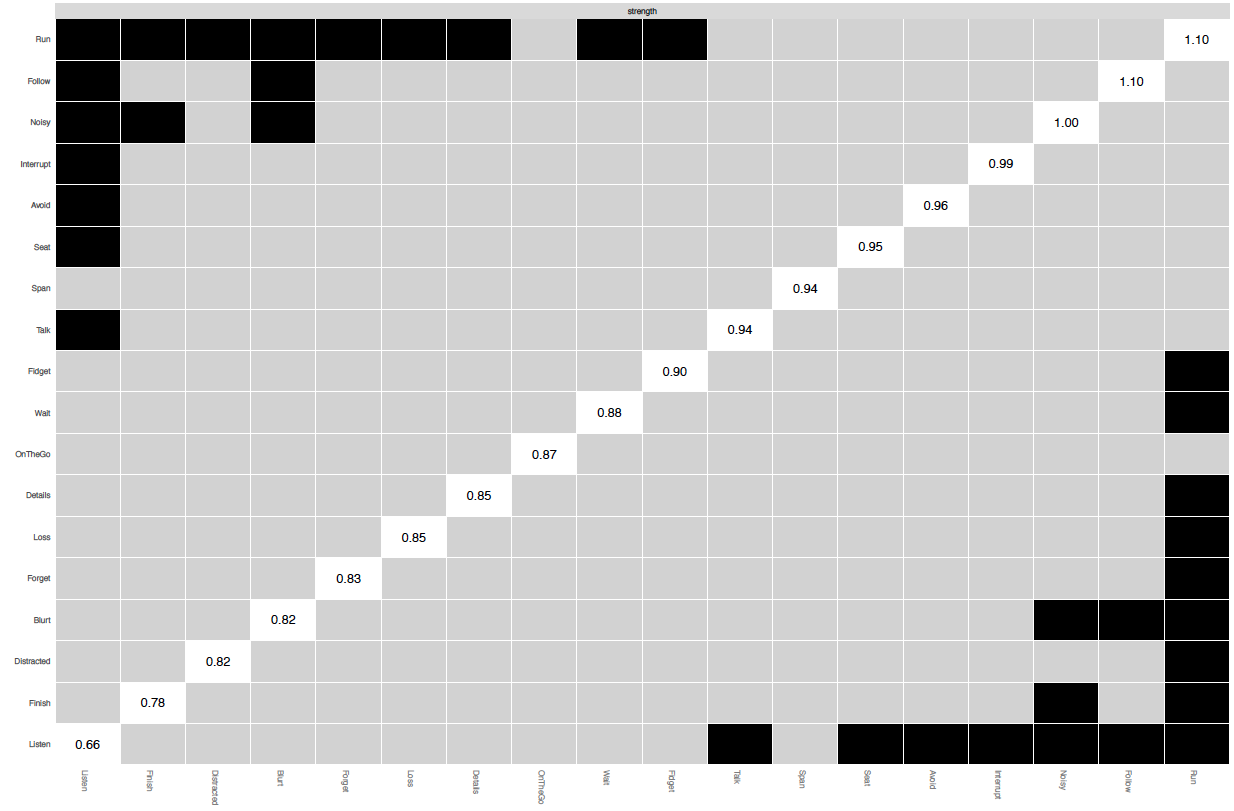


**Figure S12. Bootstrapped difference tests on the nodal strength of all the variables in the network.**

## The results for network stability and accuracy for the ADHD subtypes (ADHD-I)

**
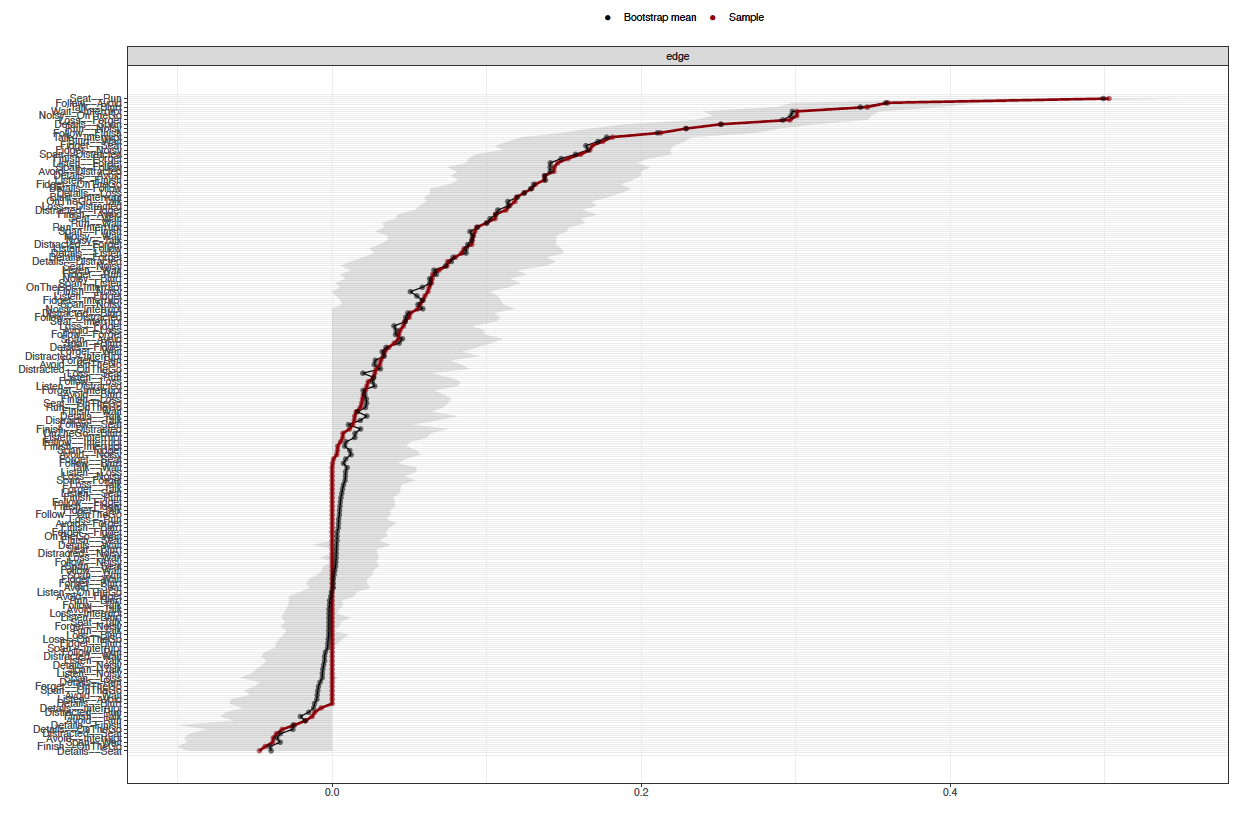
**

**Figure S13. Bootstrapped CIs of estimated edge-weights for the network.**


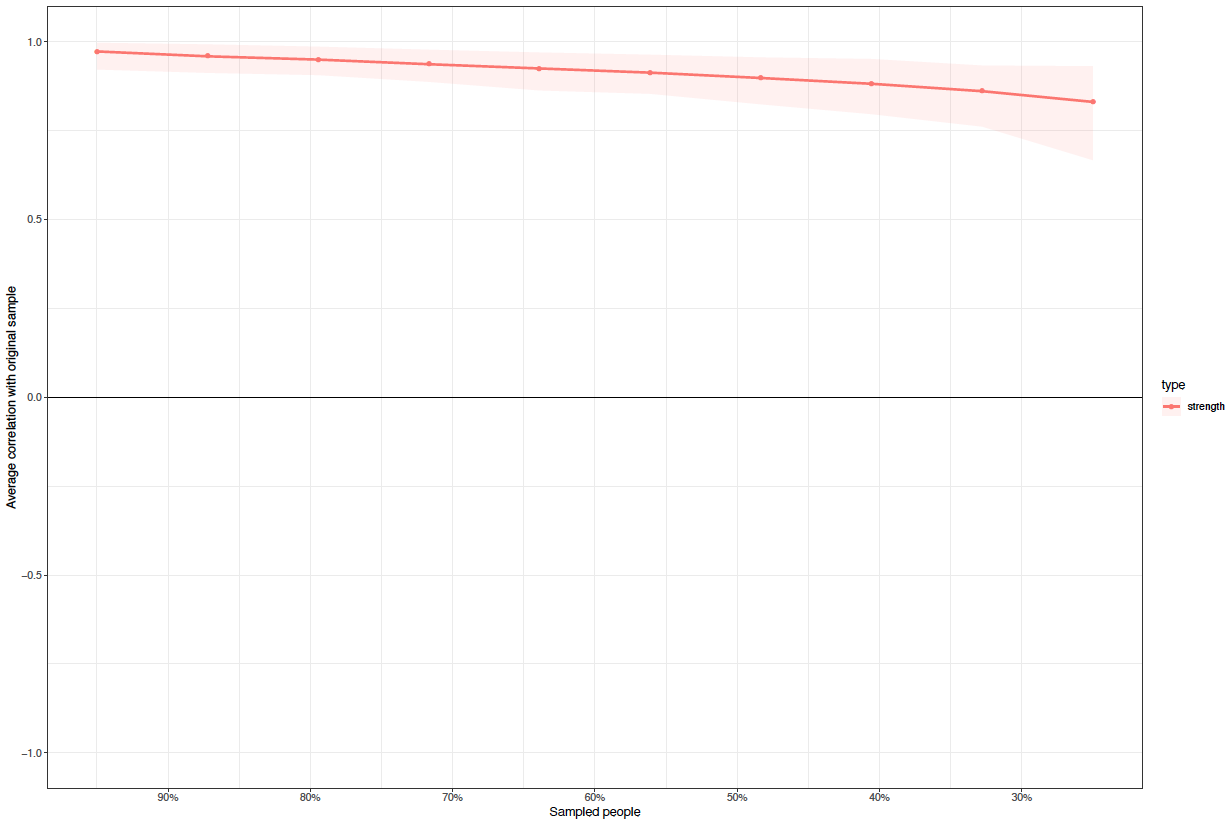


**Figure S14. Average correlations between strengths of networks estimated with sampled participants and original sample.**


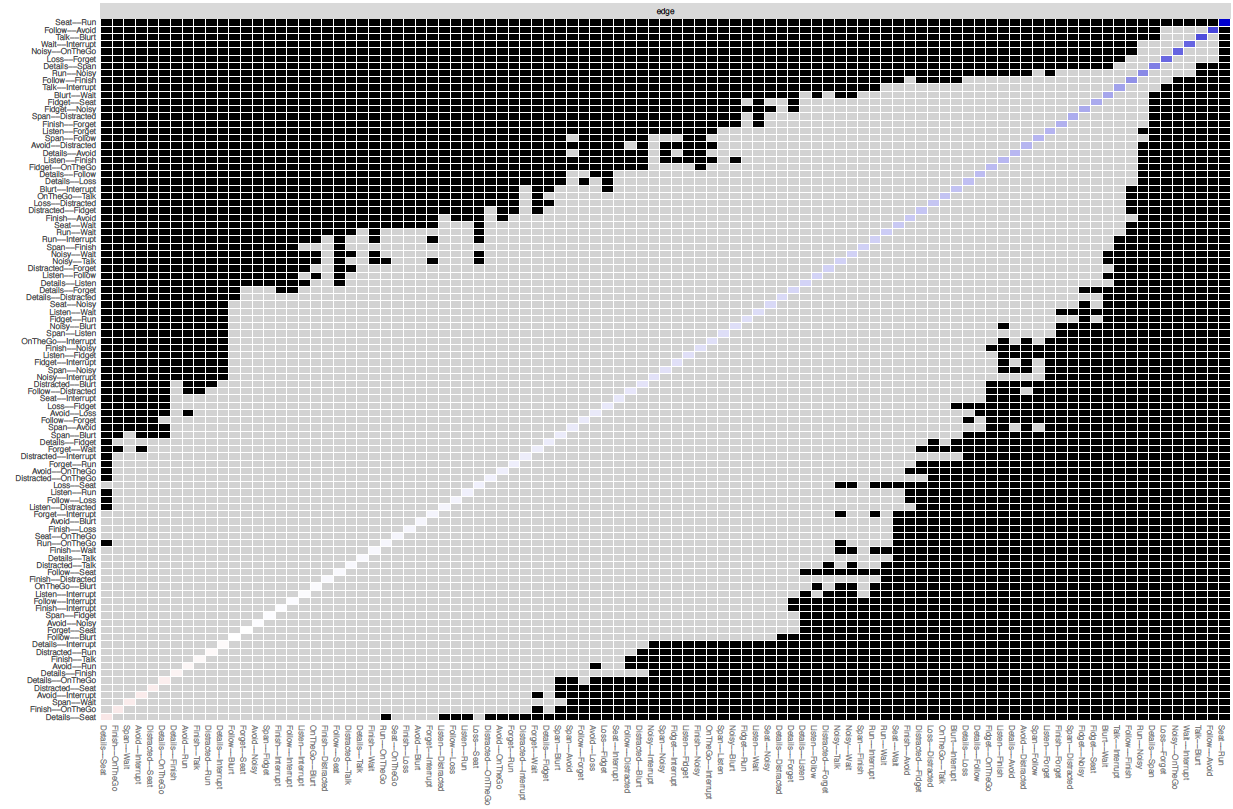


**Figure S15. Bootstrapped difference tests on the non-zero edge-weights of the estimated network.**


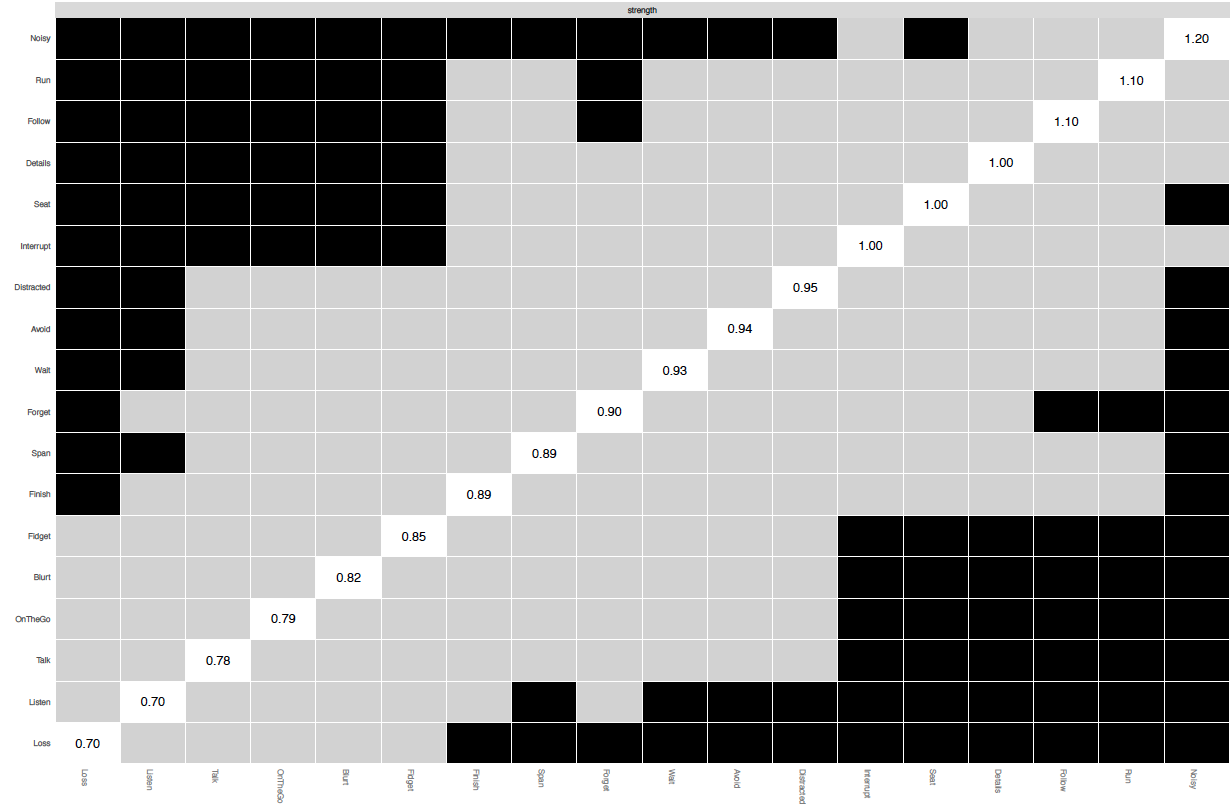


**Figure S16. Bootstrapped difference tests on the nodal strength of all the variables in the network.**

## The results for network stability and accuracy for the ADHD subtypes (ADHD-C)

**
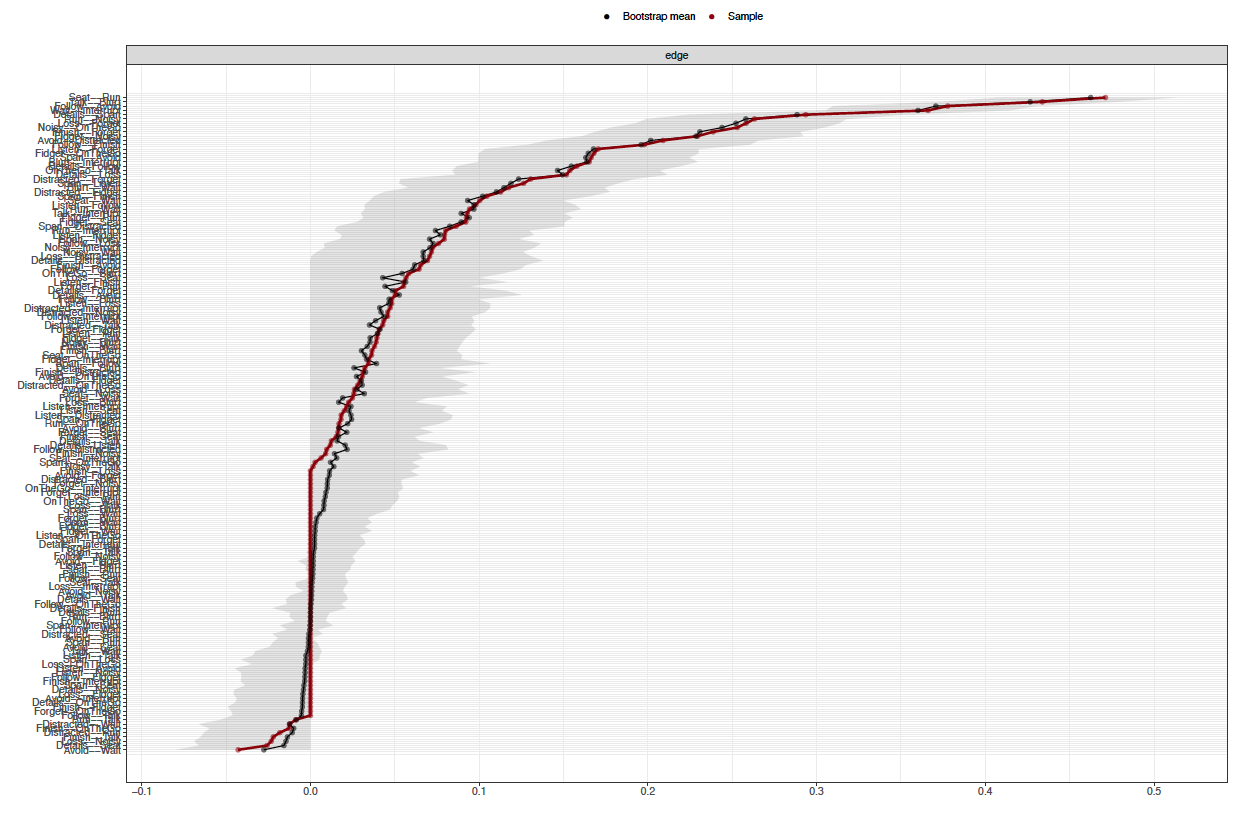
**

**Figure S17. Bootstrapped CIs of estimated edge-weights for the network.**


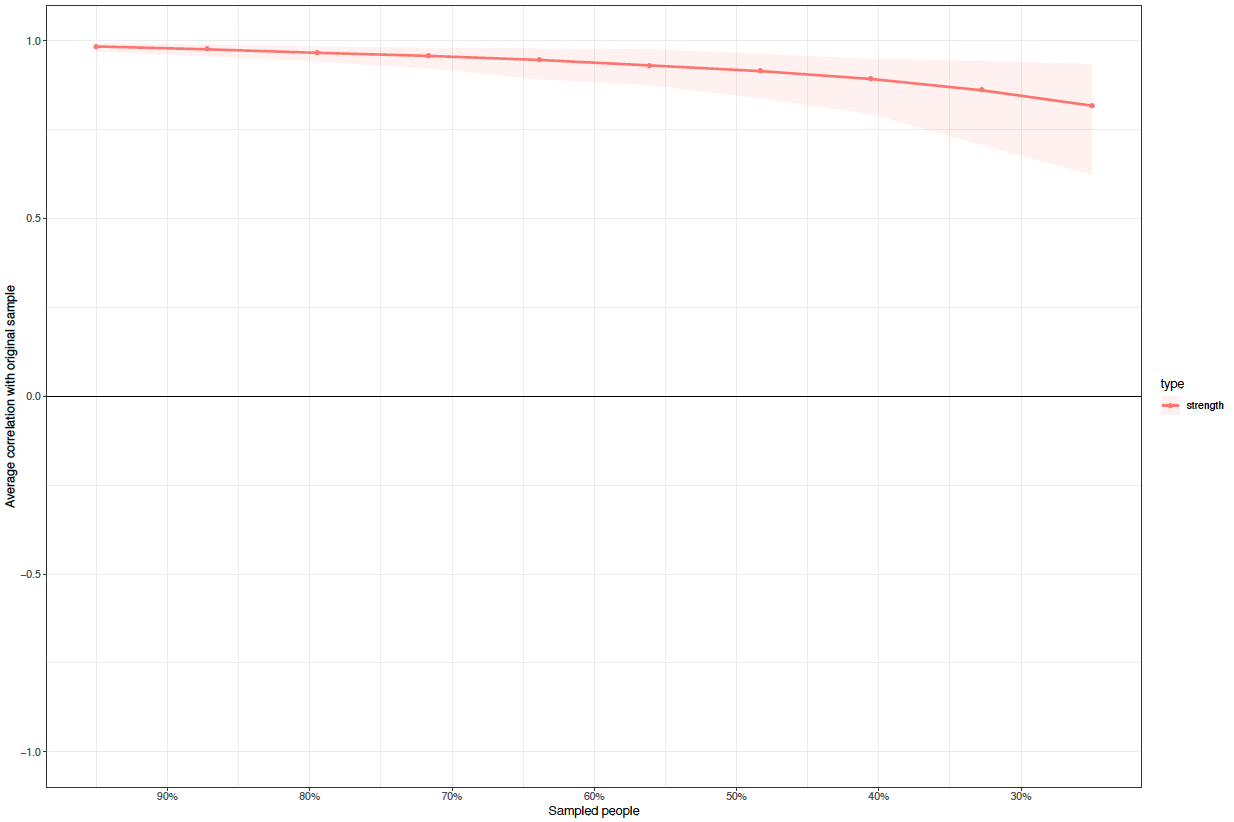


**Figure S18. Average correlations between strengths of networks estimated with sampled participants and original sample.**


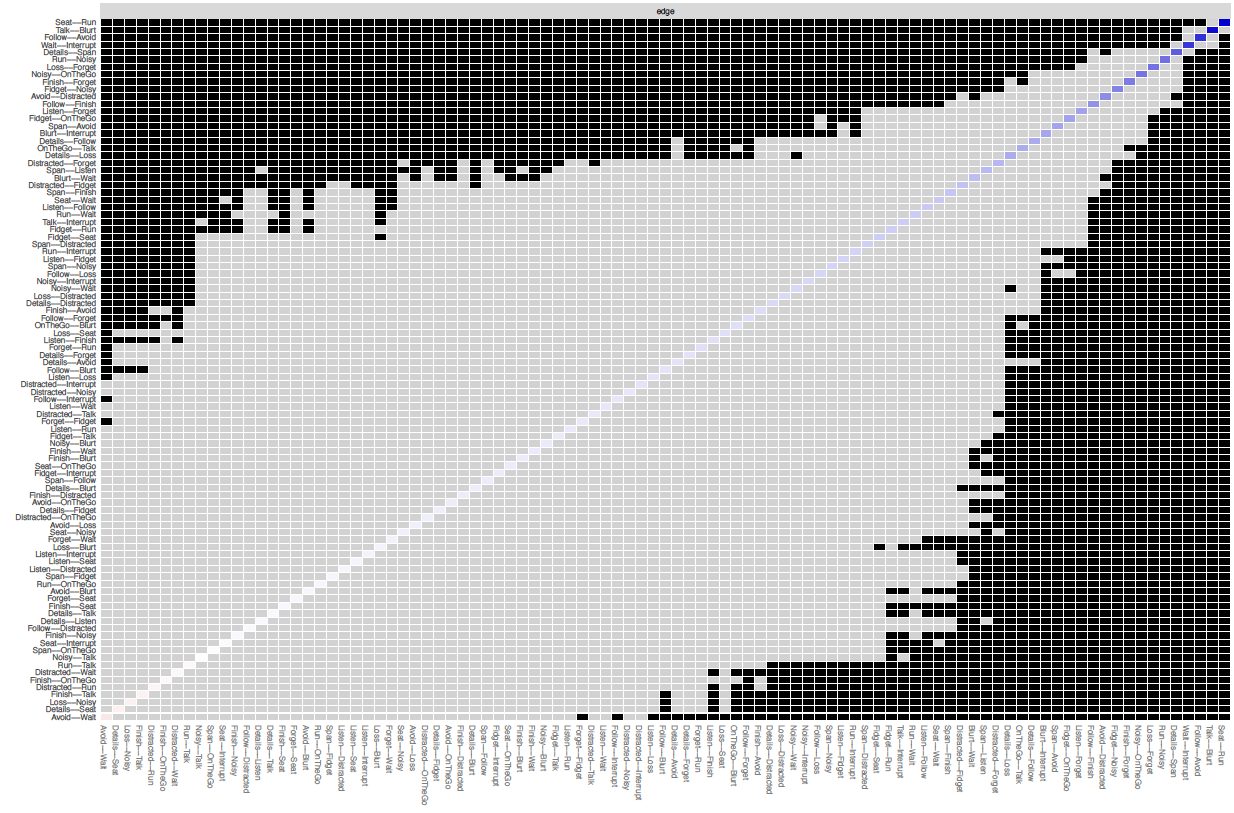


**Figure S19. Bootstrapped difference tests on the non-zero edge-weights of the estimated network.**


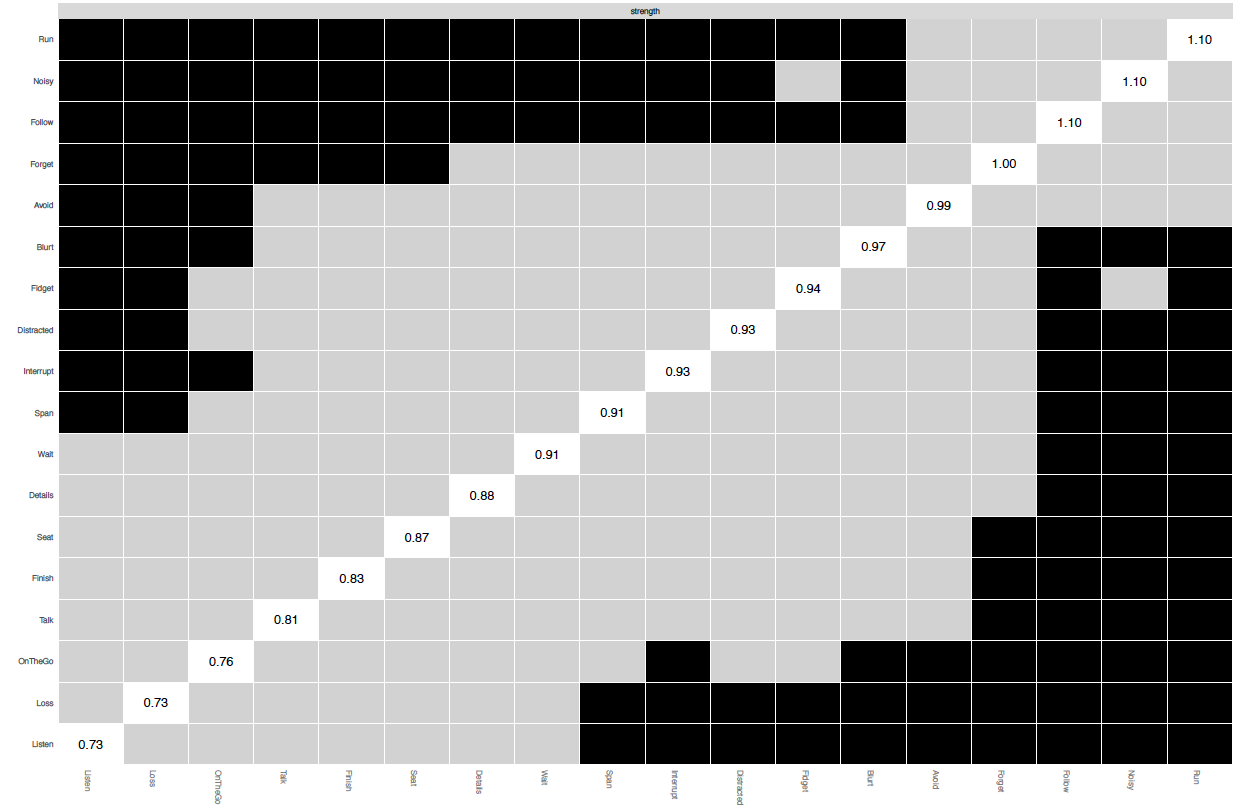


**Figure S20. Bootstrapped difference tests on the nodal strength of all the variables in the network.**

## The results for network stability and accuracy for four age subgroups

**Age group 1 (6-7 years old):**


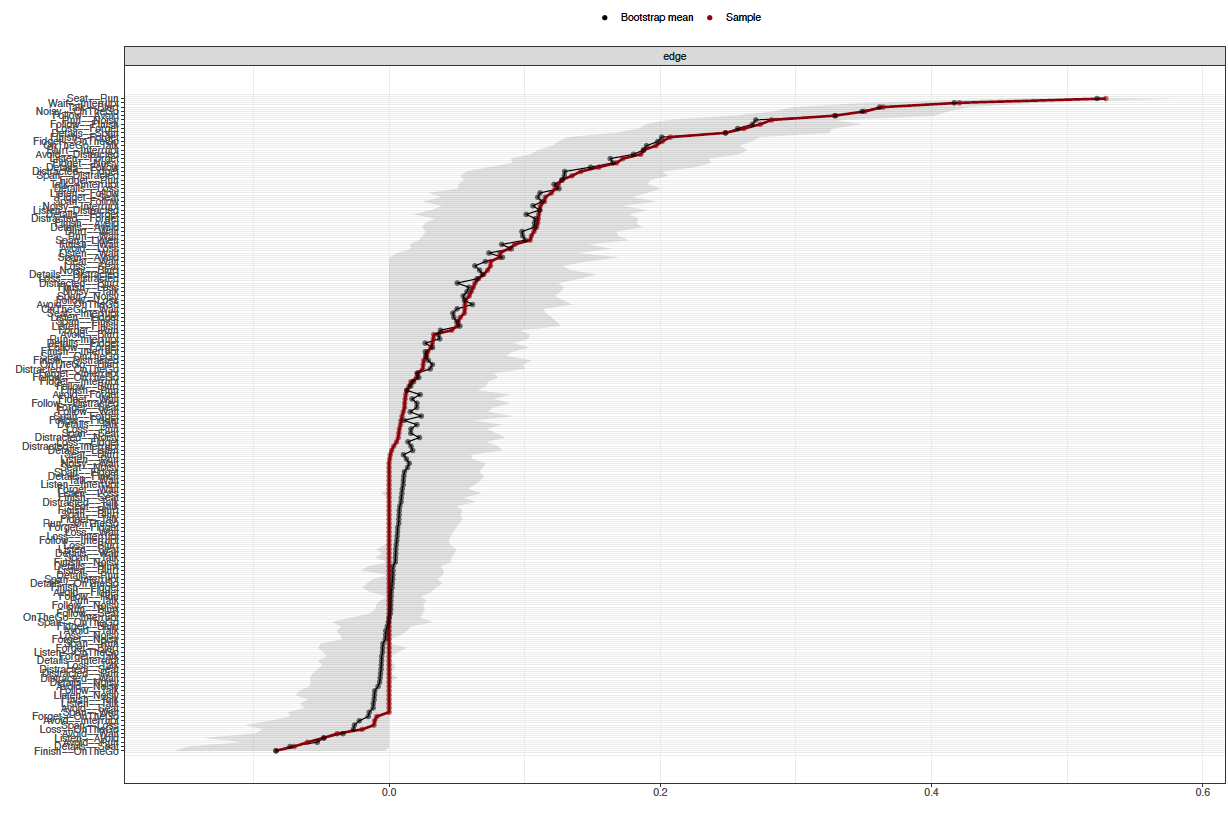


**Figure S21. Bootstrapped CIs of estimated edge-weights for the network.**


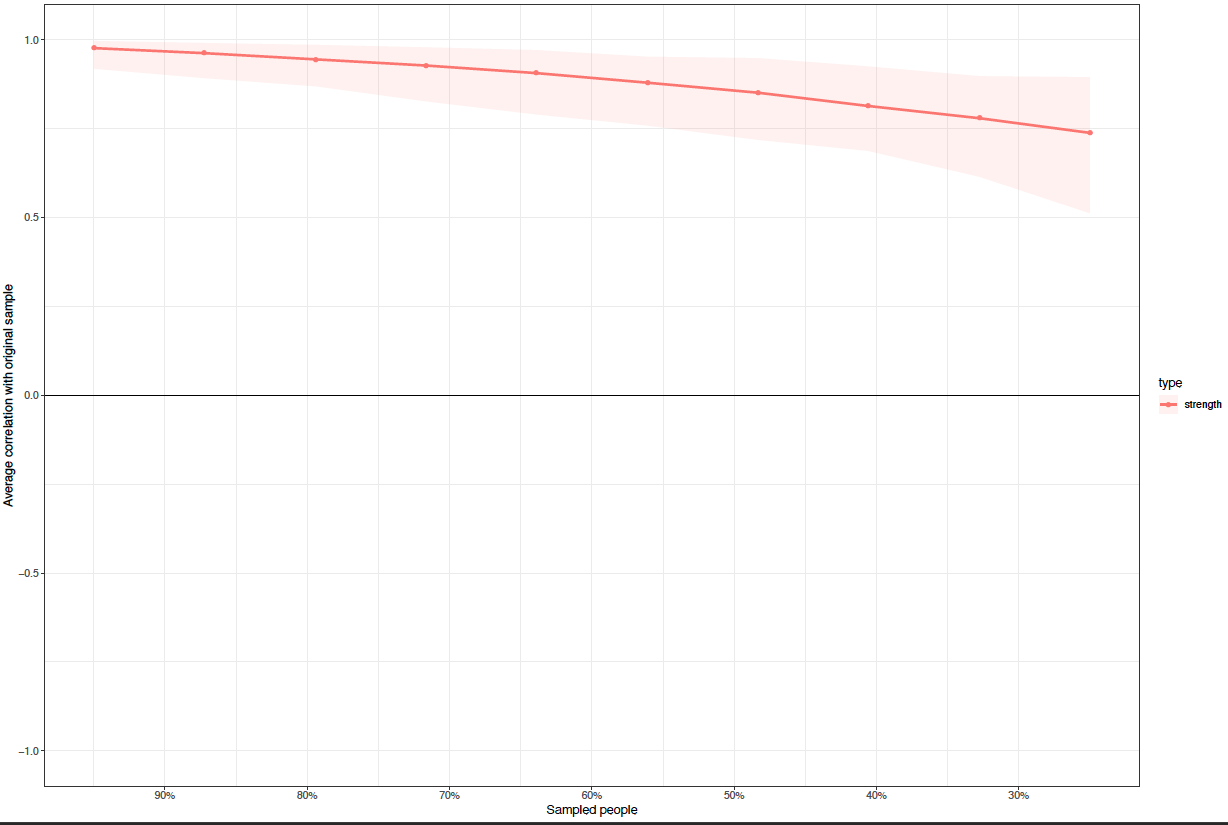


**Figure S22. Average correlations between strengths of networks estimated with sampled participants and original sample.**


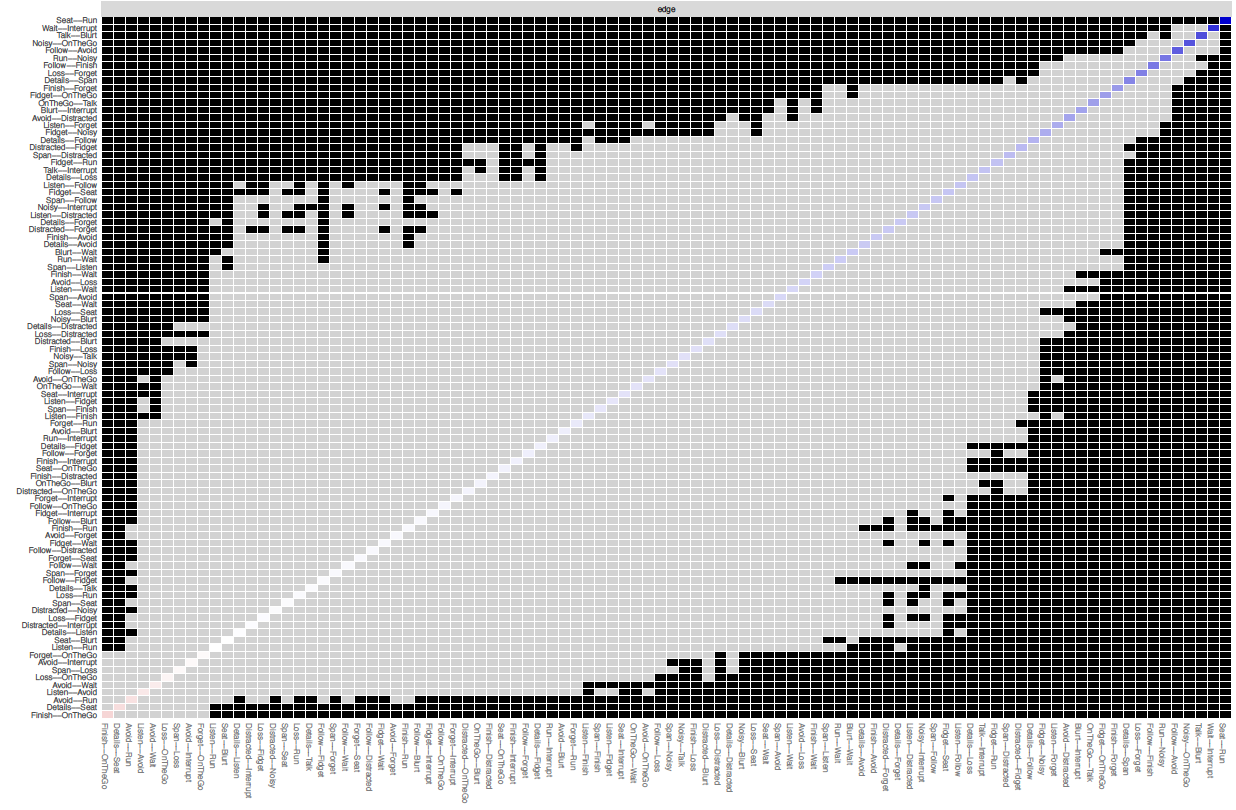


**Figure S23. Bootstrapped difference tests on the non-zero edge-weights of the estimated network.**


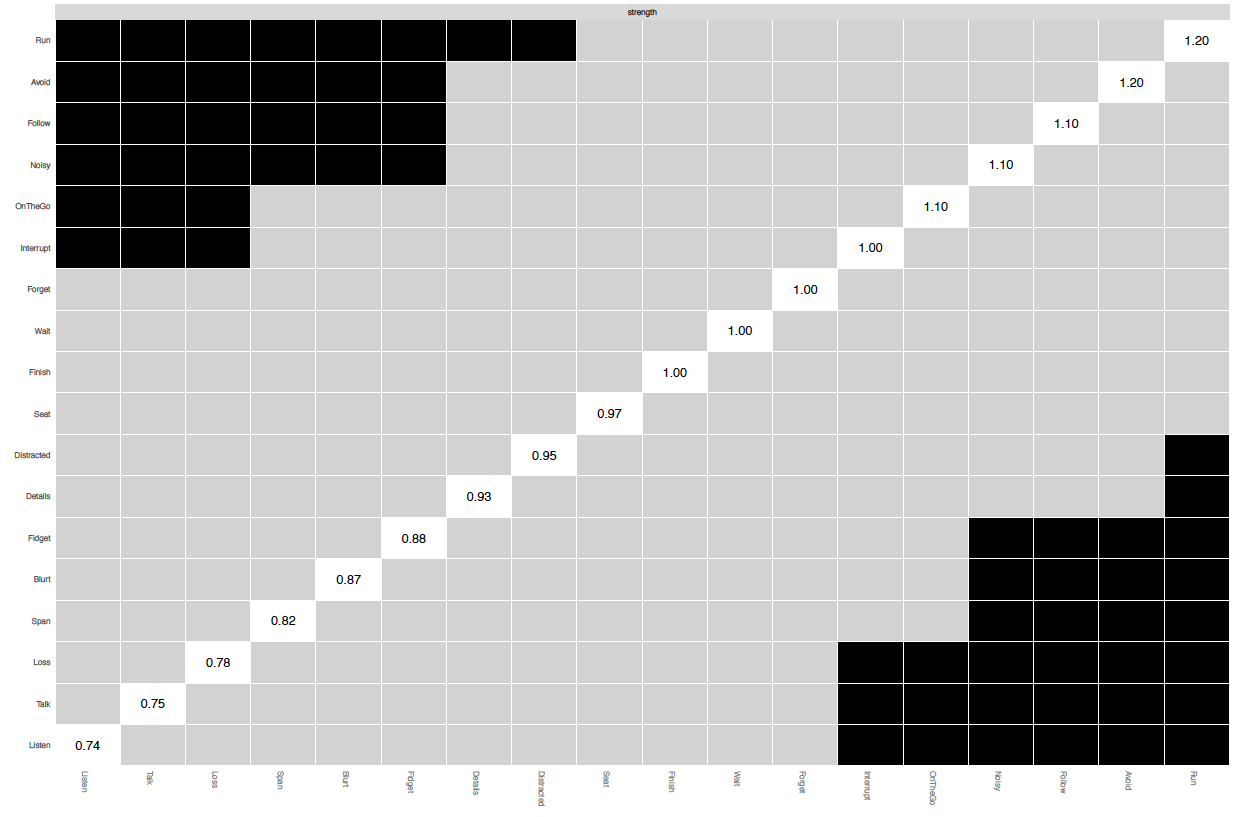


**Figure S24. Bootstrapped difference tests on the nodal strength of all the variables in the network.**

**Age group 2 (8-9 years old):**


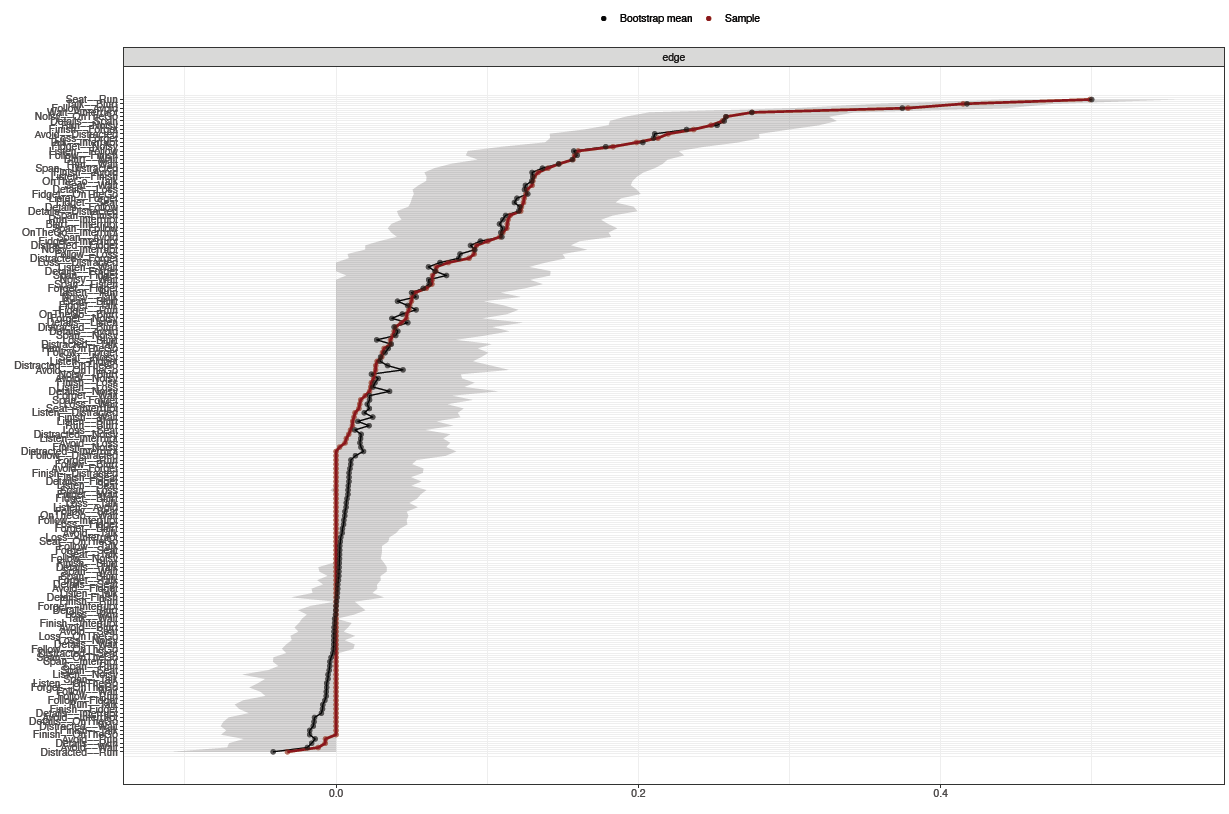


**Figure S25. Bootstrapped CIs of estimated edge-weights for the network.**


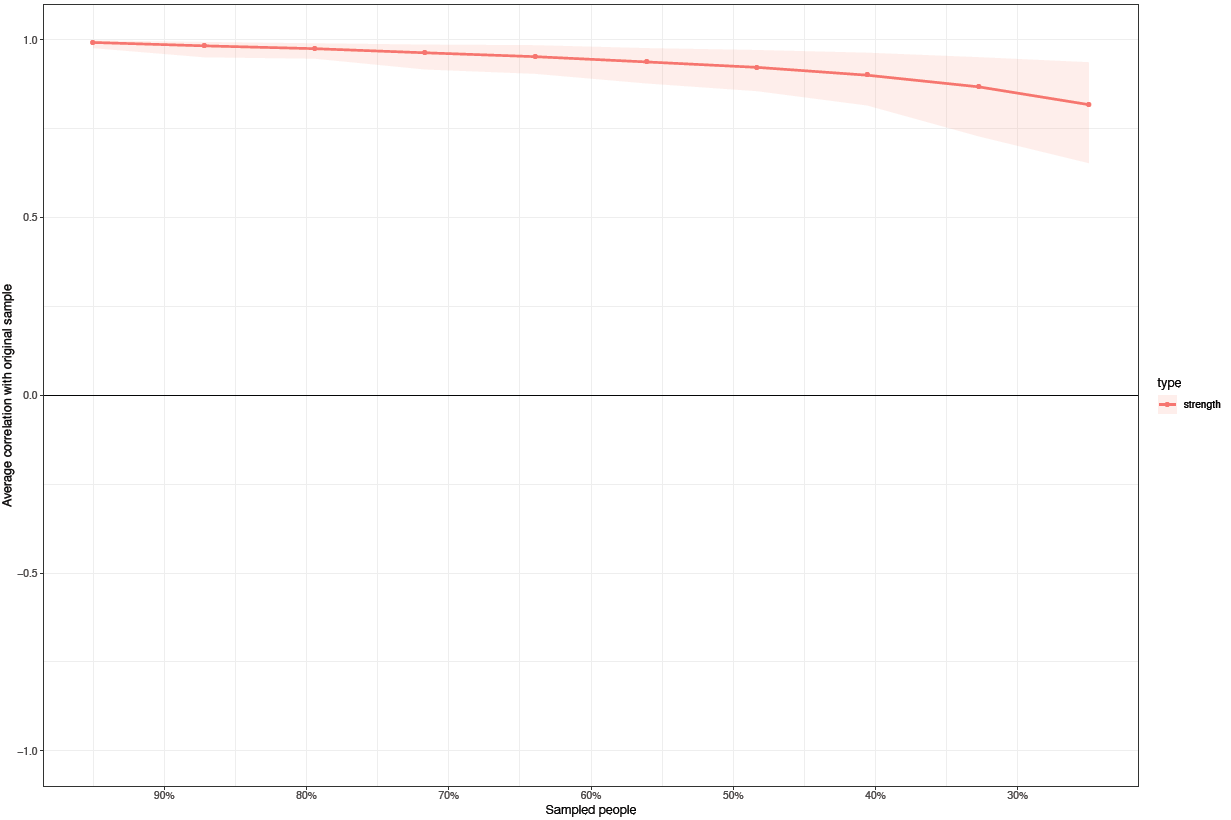


**Figure S26. Average correlations between strengths of networks estimated with sampled participants and original sample.**


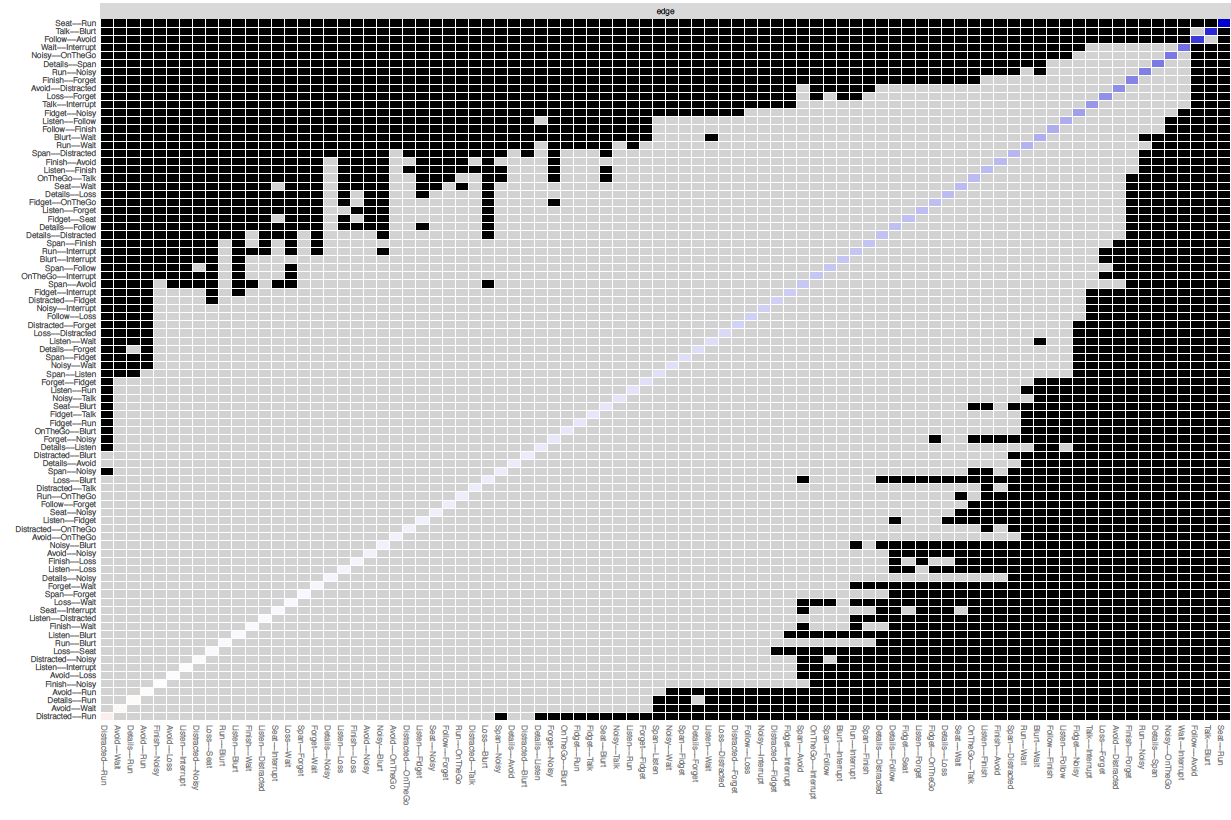


**Figure S27. Bootstrapped difference tests on the non-zero edge-weights of the estimated network.**


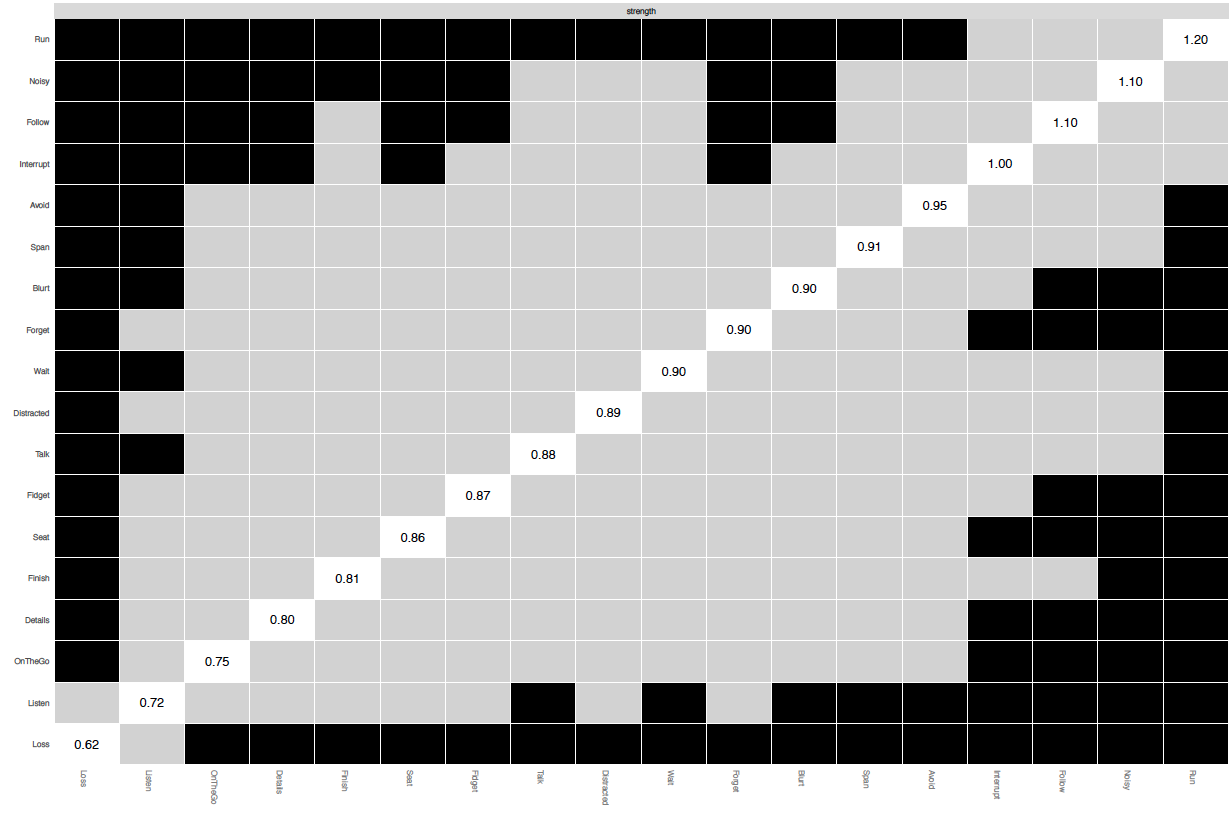


**Figure S28. Bootstrapped difference tests on the nodal strength of all the variables in the network.**

**Age group 3 (10-11 years old):**


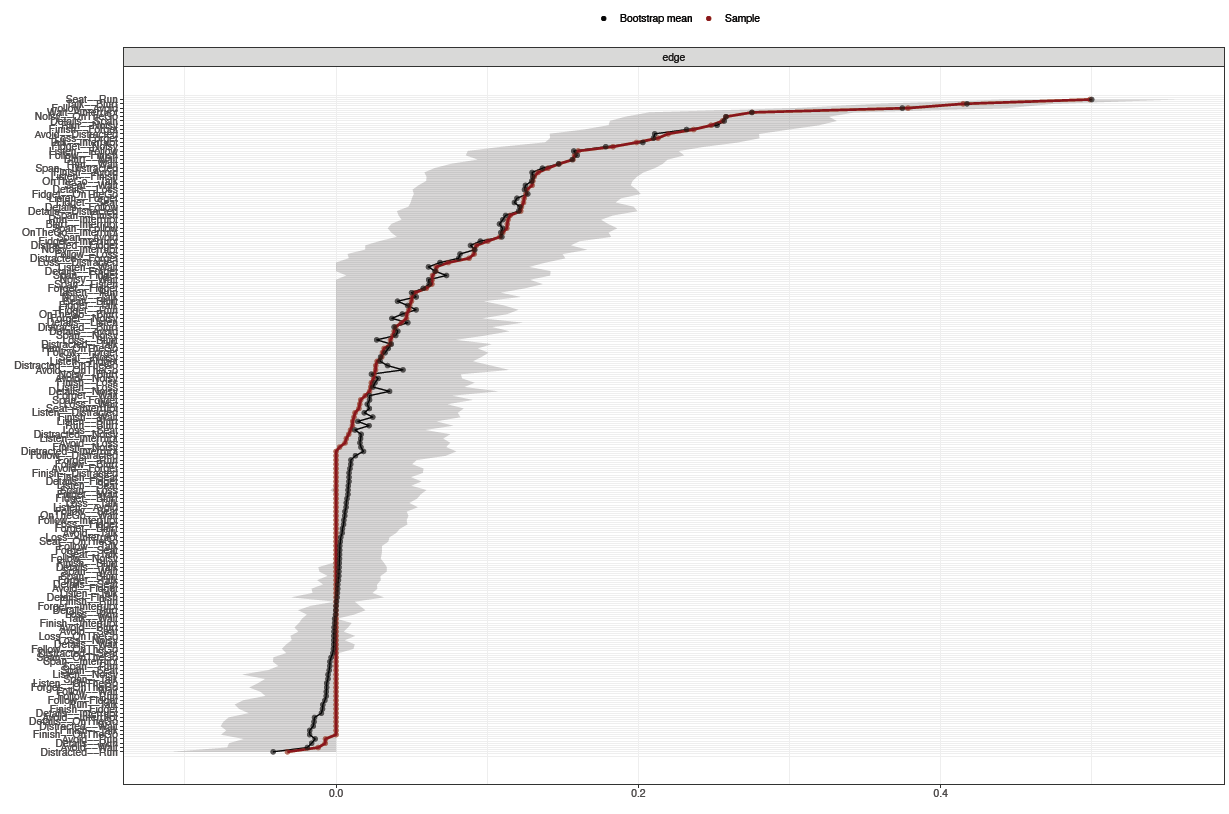


**Figure S29. Bootstrapped CIs of estimated edge-weights for the network.**


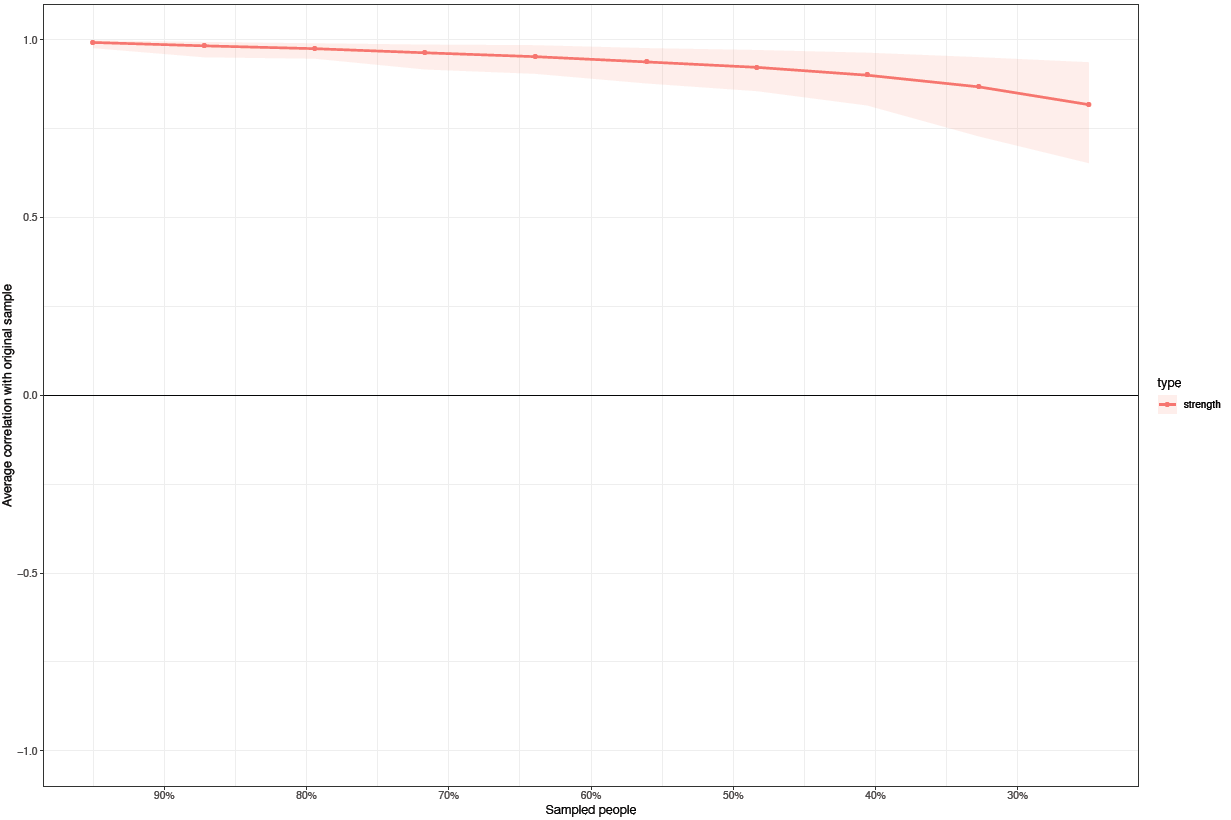


**Figure S30. Average correlations between strengths of networks estimated with sampled participants and original sample.**


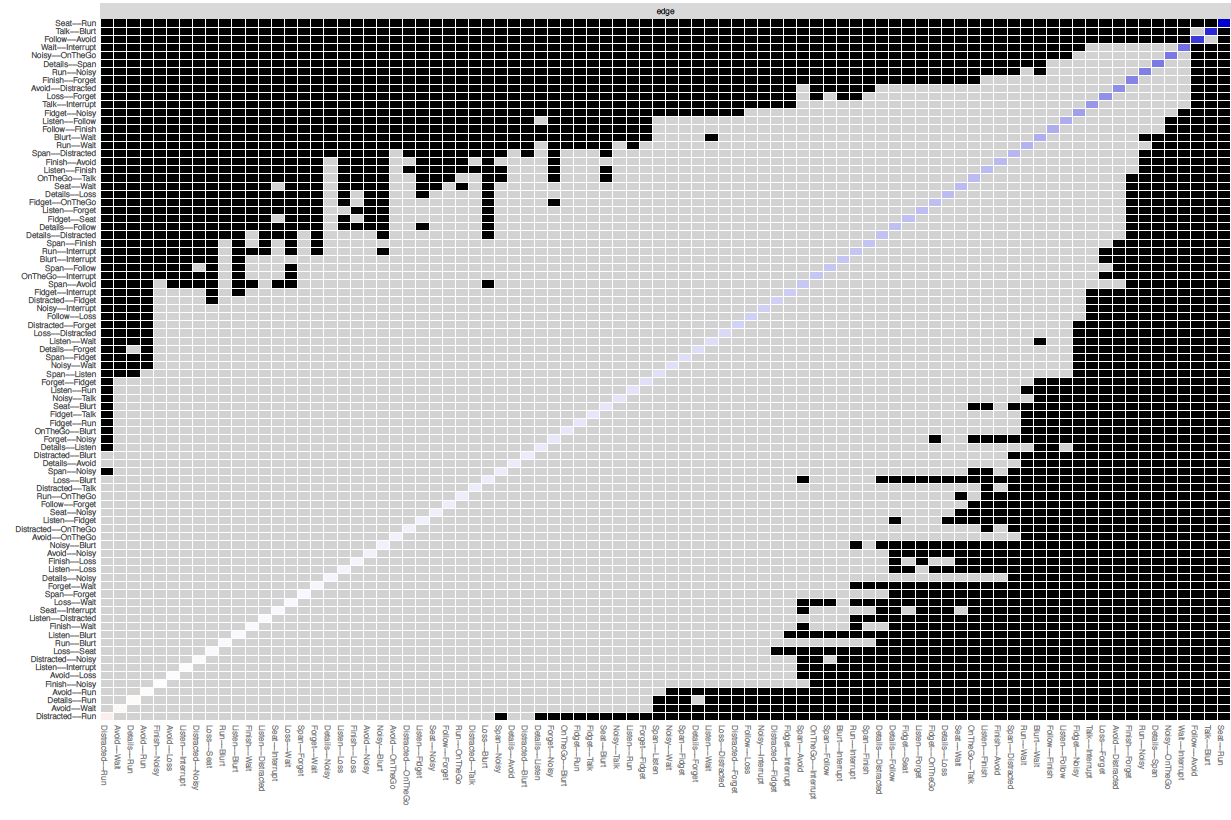


**Figure S31. Bootstrapped difference tests on the non-zero edge-weights of the estimated network.**


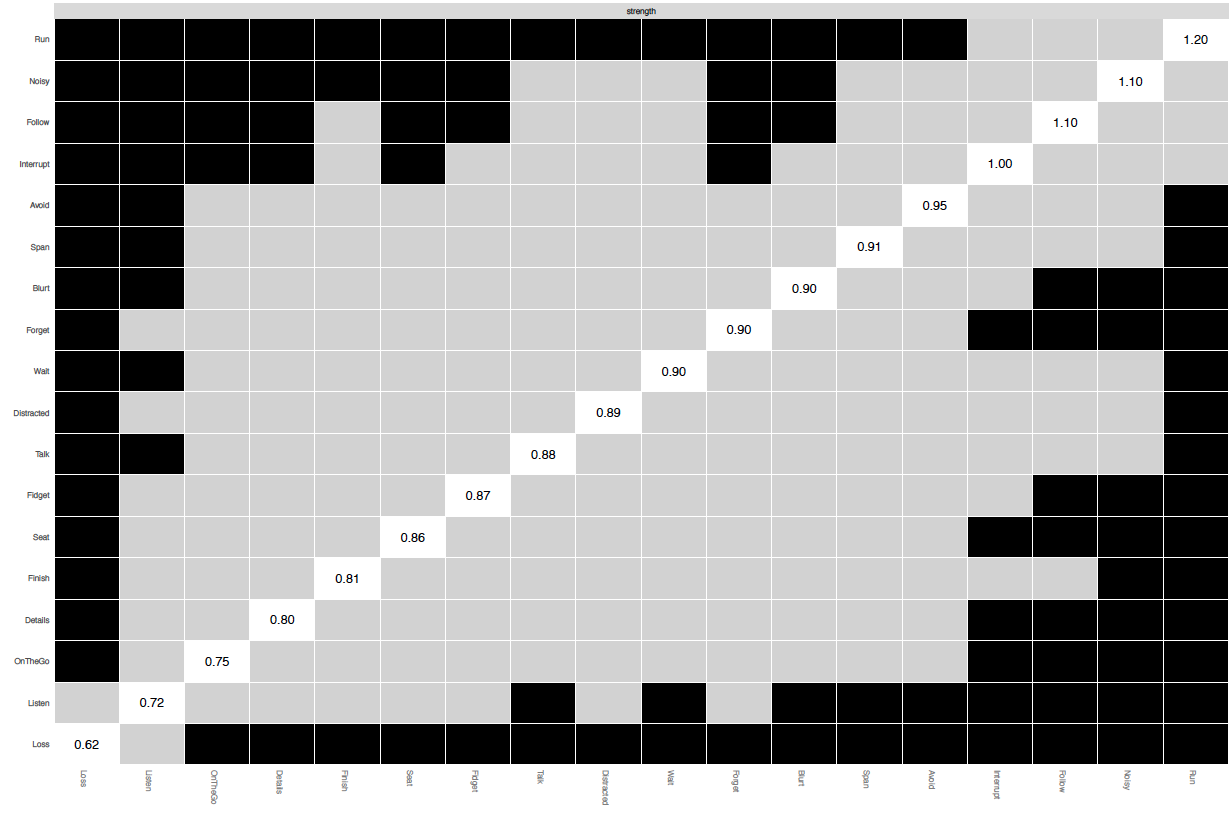


**Figure S32. Bootstrapped difference tests on the nodal strength of all the variables in the network.**

**Age group 4 (≥ 12 years old):**


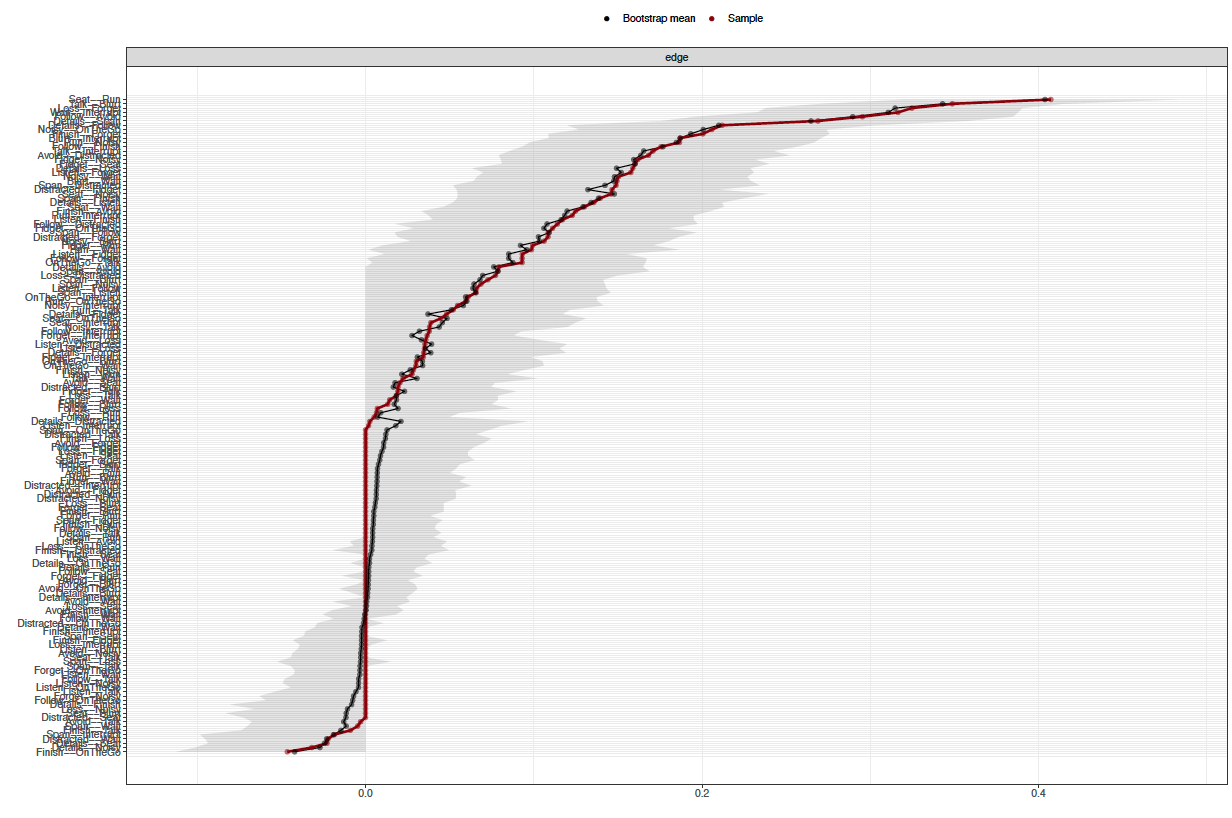


**Figure S33. Bootstrapped CIs of estimated edge-weights for the network.**


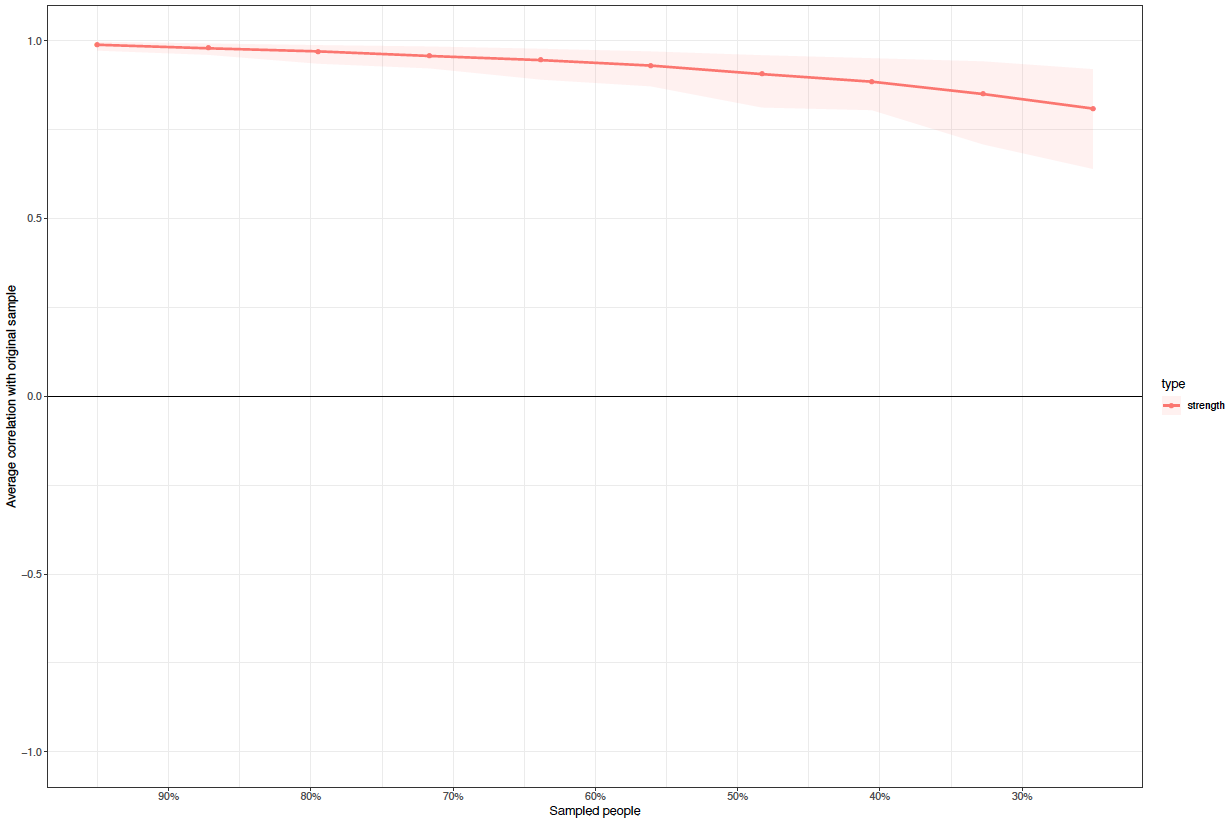


**Figure S34. Average correlations between strengths of networks estimated with sampled participants and original sample.**


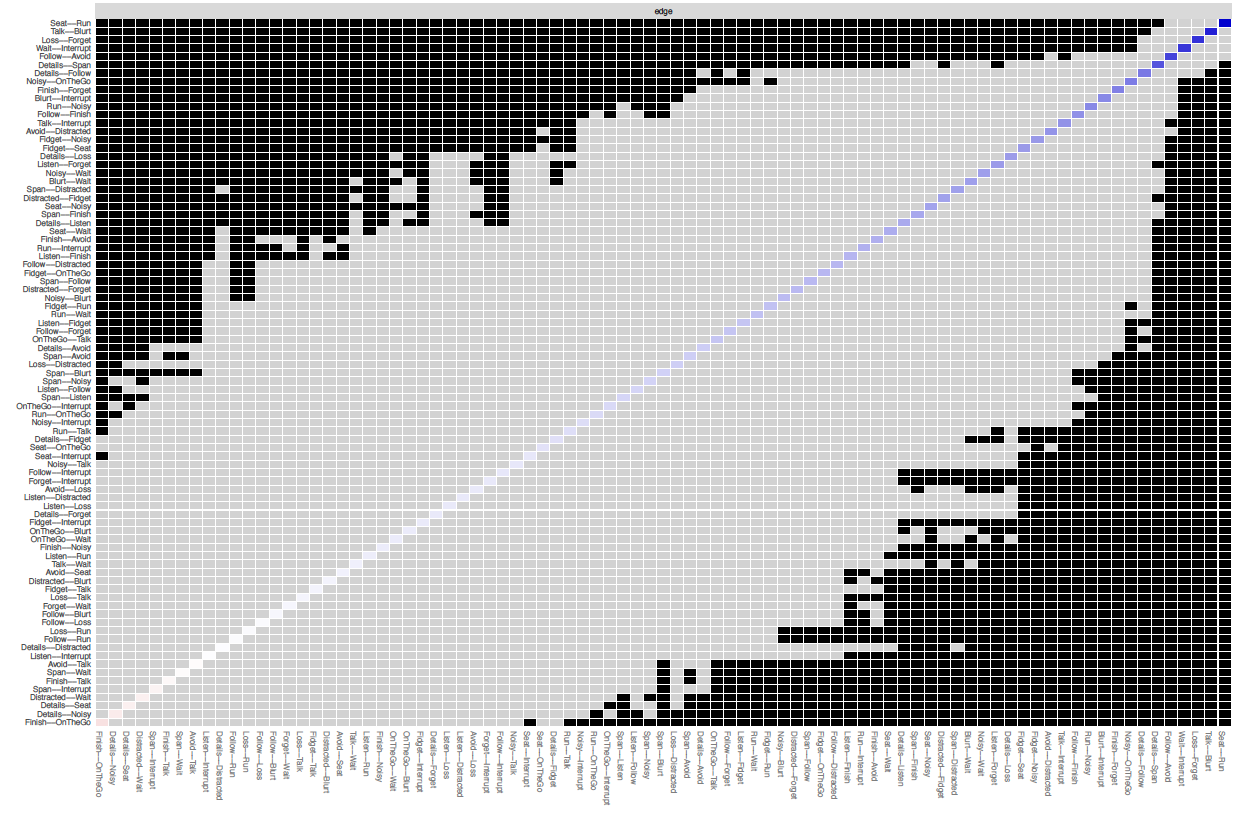


**Figure S35. Bootstrapped difference tests on the non-zero edge-weights of the estimated network.**


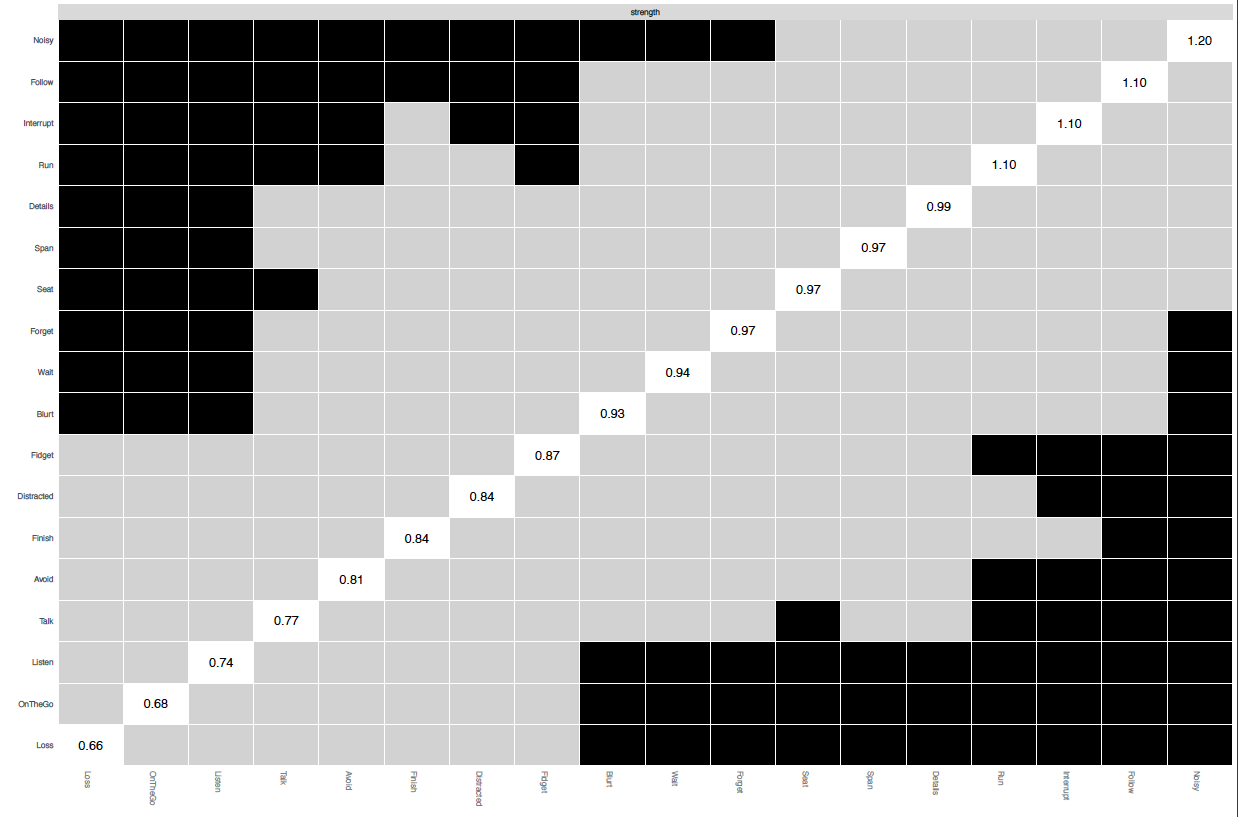


**Figure S36. Bootstrapped difference tests on the nodal strength of all the variables in the network.**
